# Variations in the Community Structure of Fungal Microbiota Associated with Apple Fruit Shaped by Fruit Bagging-Based Practice

**DOI:** 10.3390/jof7090764

**Published:** 2021-09-15

**Authors:** Punda Khwantongyim, Somying Wansee, Xi Lu, Wei Zhang, Guangyu Sun

**Affiliations:** State Key Laboratory of Crop Stress Biology in Arid Areas, College of Plant Protection, Northwest A&F University, Yangling 712100, China; kpunda@nwafu.edu.cn (P.K.); Yingku73@gmail.com (S.W.); lx12113228@163.com (X.L.); zhangweizw@nwafu.edu.cn (W.Z.)

**Keywords:** bagged apple fruit, metabarcoding, biodiversity, richness, fungal community composition, microbiome, *Malus domestica*

## Abstract

The various fungal communities that adhere to apple fruit are influenced by agricultural practices. However, the effects of fruit bagging-based management practice on the fungal microbiota are still unknown, and little is known about the fungal communities of bagged apple fruit. We conducted a study using apple fruit grown in a conventionally managed orchard where pesticide use is an indispensable practice. Fungal communities were collected from the calyx-end and peel tissues of bagged and unbagged fruit and characterized using barcode-type next-generation sequencing. Fruit bagging had a stronger effect on fungal richness, abundance, and diversity of the fungal microbiota in comparison to non-bagging. In addition, bagging also impacted the compositional variation of the fungal communities inhabiting each fruit part. We observed that fruit bagging had a tendency to maintain ecological equilibrium since Ascomycota and Basidiomycota were more distributed in bagged fruit than in unbagged fruit. These fungal communities consist of beneficial fungi rather than potentially harmful fungi. Approximately 50 dominant taxa were detected in bagged fruit, for example, beneficial genera such as *Articulospora*, *Bullera, Cryptococcus*, *Dioszegia*, *Erythrobasidium*, and *Sporobolomyces*, as well as pathogenic genera such as *Aureobasidium* and *Taphrina*. These results suggested that fruit bagging could significantly increase fungal richness and promote healthy fungal communities, especially the harmless fungal communities, which might be helpful for protecting fruit from the effects of pathogens. This study provides a foundation for understanding the impacts of bagging-based practice on the associated fungal microbiota.

## 1. Introduction

Apple (*Malus domestica* Borkh.) is an important rosaceous species and is the most economically and culturally important fruit worldwide. Its cultivation is fostered in many parts of the world, with an annual production surpassing 86 million metric tons, making it the third most cultivated fruit worldwide (http://faostat.fao.org (accessed on 5 May 2021)). There has been an estimated reduction in apple production of 12–15% in the annual apple harvest [1]. Climate change (e.g., hailstorms and heavy rain) and fruit damage caused by insects and diseases are the key issues causing economic yield losses [1,2,3]. Pesticides have been widely used in the main production regions of China to protect apples from the threat of pests. However, pesticide residues, including apple fruit pesticide residues, can accumulate and pose a threat to the health of various agroecosystems. Therefore, to prevent losses from abiotic and biotic factors, several good agricultural practices (GAP) have been developed for high-quality production worldwide [4,5]. Fruit bagging is one of the cultivation techniques used for GAP to improve fruit quality, which has been used extensively in many countries [6,7,8,9]. Over past two-decades, fruit bagging has become an essential management practice for apple production in China. Bagging can produce high-quality fruit, and it has been found to increase the solid soluble content [10]. It can reduce anthocyanin biosynthesis, promote fruit coloration [10,11], and reduce immediate dietary risks [12,13]. Moreover, this management practice can be used as a physical barrier to prevent damage from birds [4,6,14], from insects such as *Carposina niponensis*, *Adoxophyes orana*, [6,14,15], and from diseases such as ring rot (*Botryosphaeria* spp.) and bitter rot (*Colletotrichum* spp.) [6,15,16,17]. Some diseases, however, appear with the changes to the condition inside the fruit bags. Wang et al. [15] reported a brown spot in apples due to bagging, caused by high humidity inside the bag, thereby promoting brown spot disease [18].

The apple is a widely grown, highly essential crop and naturally hosts a reservoir of microorganisms [19,20,21,22]. Since microorganisms are alive as part of the agricultural environment, they interact with plant hosts, leading to ecological equilibrium, and are significant components of biodiversity in agroecosystems [23]. The endosphere and phyllosphere are extensive microenvironments of microorganisms such as endophytes and epiphytes [24,25]. Microbial populations include symbionts, antagonists, and pathogens that can be beneficial or harmful to their hosts. Microorganisms can promote plant growth, enhance nutrient uptake, and increase the ability to tolerate abiotic stresses such as drought and salinity [26]. On the other hand, some fungal microorganisms especially those belonging to the Botryosphaeriaceae and Sclerotiniaceae families, are considered latent or opportunistic pathogens capable of causing diseases when plants are rapidly stressed [27,28].

Thus far, the interactions between apple and fungal populations have been studied extensively in bud [29], fruit [19,30,31], leaf [29,32], and root [33] based on the traditional tissue isolation. However, it is difficult to obtain slower-growing fungi by culturing. Some fungi are unable to grow on the tested media. Moreover, the most abundant microorganisms identified are estimated to represent only a tiny fraction of the total existing microorganisms [34]. As a consequence, the fungal microbiota of apple or other fruits are poorly understood. In particular, there is little available information regarding the complex interactions between microbial populations and their habitat. Thus, next-generation sequencing (NGS) has been proven to overcome the limitations of traditional approaches [35,36], providing new insights on fungal microbiota and aiding in our understanding of obscure microbes in nature in different environments [20,21,37,38,39,40] and the structure of such fungal communities in complex ecosystems. However, the potential of NGS deep sequencing (e.g., Illumina sequencing) has been limited in its reliability by systemic errors in PCR and sequencing [41]. Oversampling or duplication could occur during gene target enrichment by either PCR amplification or Illumina sequencing [41]. In addition, some fragmented genomic DNA can be amplified into multiple identical ones. Repeated reads from these PCR duplicates may yield false positive repeats. Thus, PCR amplification bias can introduce different duplication levels in different genes, which is the main source of inaccuracy. Deep sequencing combined with unique identifier (UID) barcoding is a technique developed to address these issues and detect rare mutations [42,43] to obtain accurate gene expression information. 

In general, NGS technology has become an admirable tool for studying the microbial communities of plant hosts [21,23,35,36,44,45,46], including apples [20,21,23,39,47]. For example, Liu et al. [21] investigated the microbiota of different apple rootstock/scion combinations using high-throughput amplicon sequencing. The results showed greater diversity of fungi than that of bacteria, and the microbial communities of closely related cultivars of two scions were more similar to each other than to others derived from a distantly related cultivar. In addition, thus far, many studies have reported an association between fungal communities found in and on apples affected by farming management or cultivation practices (e.g., conventional, integrated, and organic practices) [19,20,23]. A fungal community consists of fungal composition, abundance, and diversity in apple fruit, which is closely related to fruit development, fruit management practices, and quality control [19,20,23,48]. Keys management practices in apple production have been reported, particularly fruit bagging, which involves the control of apple pests [14,48,49]. However, no study has yet reported on the microbial community in apple production under bagging-based management practice. 

Compared to the traditional practice of using pesticides alone in the management of farm systems, fruit bagging protects from pesticide accumulation and changes the microenvironment conditions, such as light exposure, wetness, and temperature and also alters the metabolism of fruit and secretions on the fruit’s surface. All of these factors affect the fungal community. However, the effects of fruit bagging on apple fruit inhabited by a fungal community are still not clear. Therefore, in this study, fungal populations associated with bagged and unbagged apple fruit were investigated using UID barcoding next-generation sequencing based on ITS regions. Our aims were: (1) to identify the differences in fungal community structure and diversity between different practices, including bagging- and non-bagging-based practices; (2) to investigate the impacts of bagging-based practice on the fungal community in different niches, including calyx-end and peel tissues; and (3) to assess the impact of bagging-based management practice on fungal communities regarding benefits in an agroecosystem.

## 2. Materials and Methods

### 2.1. Orchard Site and Apple Sample Collection 

The study was carried out on the apple fruit of approximately 15-year-old trees of the Fuji variety, which were obtained from the orchard in Yintai District, Tongchuan City, Shaanxi Province of China. We used two-layer commercial bags to cover over fruit during the production practice. Aside from bagging, apples were treated by management programs (Table 1) to protect apples from diseases and insects during bagging-based management practice. The sites of non-bagging and bagging were labeled as R3 and R5, respectively. Healthy mature apple fruit was collected randomly from each site, gathered in a sterile plastic bag, and transported to the laboratory to be stored at 0 °C for further processing.

### 2.2. Sampling

The twelve samples of peel and calyx-end tissues were separated from the bagged and unbagged apple fruit, with each part of the apple fruit weighing approximately 1 g. In the whole process of sampling, we handled the apple fruit with gloved hands. We named the apple calyx-end and peel parts derived from fruit of non-bagging-based management practice as CR3 and PFR3, respectively, while apple calyx-end and peel tissues from bagging-based management practice were labeled CR5, and PFR5, respectively. All of these tissue fragments were cut to represent the CR3, CR5, PFR3, and PFR5 samples and immediately frozen in liquid nitrogen before being stored in 2 mL vials at −80 °C for further processing. 

### 2.3. Bioinformatic Analysis

High-throughput sequencing was performed by SeqHealth Tech Co, Ltd. Wuhan, China. Briefly, the genomic DNA of the collected samples was extracted using the CTAB-based modified method [50]. The concentrations of DNA were measured using a NanodropTM OneC spectrophotometer (Thermo Fisher Scientific Inc., Waltham, MA, USA), and DNA integrity was assessed using standard denaturing agarose gel electrophoresis. Since we targeted the Internal Transcribed Spacer (ITS) regions of the ribosomal RNA gene cluster, ITS1 was extended by eight random bases UID to obtain more accurate gene expression. All primers were performed followed by ligation of the P5 and P7 adaptors to facilitate the barcoded library for Illumina sequencing. After total DNA extraction, the required DNA fragments were amplified by PCR with ITS gene-specific primers extended with UID and adaptors, followed by bead purifications. The purified products were subjected to target enrichment for subsequent PCR. Subsequently, the UID barcode templates were re-amplified and bead purifications were performed to ensure that sufficient adaptor dimers were removed. The required DNA libraries were pooled and sequenced on Illumina Novaseq 6000 with 2 × 300 bp chemistry.

Raw sequencing data were first filtered using the Trimmomatic bioinformatics software (Version 0.36) to ensure the quality of subsequent information analysis. Clean reads were further treated with in-house scripts to eliminate duplication bias introduced in the library preparation and sequencing. Briefly, clean reads were first clustered according to the UID sequences. Reads with the same UID sequence were grouped in the same cluster. Reads in the same cluster were compared with each other by pairwise alignment, and then reads with a sequence identity above 95% were extracted into a new sub-cluster. After all the sub-clusters were generated, multiple sequence alignment was performed to obtain a consensus sequence for each sub-cluster. After these steps, all errors and biases introduced by PCR amplification or sequencing were eliminated.

Subsequently, 300 bp paired-end reads were merged using FLASH software (FLASH: Fast length adjustment of short reads) to improve genome assemblies [51]. These high quality reads were clustered into operational taxonomic units (OTUs) using a threshold of 97% similarity for fungi against the UNITE-ITS database (https://www.mothur.org/wiki/UNITE_ITS_database (accessed on 20 April 2020)) [52]. The OTU taxonomy was simplified by removing rare OTUs (≤0.001% of the total sequences) to reduce the complexity of the subsequent analysis [53]. Moreover, rarefaction was used to an even sequencing depth for normalizing the OUT table. Qualitative and quantitative information of the fungal rarefied OUT table was used for subsequent analysis. 

### 2.4. Statistical Analysis

The abundance of rarefied OTUs across all samples was calculated in QIIME using the non-parametric Kruskal–Wallis/FDR test. The relative abundance of fungal genera that exhibited significant (*p* < 0.05) differential abundance across all samples was shown in a heatmap using the heatmap.2 function in the gplots package of R software [54].In addition, the richness and diversity of the fungal communities were first explained by plotting rarefaction curves of the OTU table data. The rarefied OTU tables served as an input matrix for the fungal composition assessment and diversity analyses (alpha (α) and beta (β) diversity) and were calculated according to the statistics in QIIME. Alpha diversity indices were selected for calculation in this study, including the Chao1, Shannon, and Simpson indices. All of these alpha diversity metrics were calculated as follows:

- Chao1 estimator [55]:
  (1) $$S_{chao1}=S_{obs}+\frac{n_{1}\left(n_{1}-1\right)}{2\left(n_{2}+1\right)}$$
  where *n*_1_ and *n*_2_ are the count of one sequence (i.e., singletons) and two sequences (i.e., doubletons), respectively, and *S_obs_* is the observed number of species.
- Shannon estimator [56]:
  (2) $$H_{shannon}=-\overset{S_{obs}}{\underset{i=1}{\sum }}\frac{n_{i}}{N}ln\frac{n_{i}}{N}$$
  where *S_obs_* is the number of observed OTUs, *N* = the total number of individuals in the community, and *n_i_* is the number of individuals in OUT*i*.
- Simpson estimator [57]:
  (3) $$D_{simpson}=\frac{\sum_{i=1}^{S_{obs}}n_{i}\left(n_{i}-1\right)}{N\left(N-1\right)}$$
  where *S_obs_* is the number of observed OTUs or species in the community, *N* = the total number of individuals in the community, and *n_i_* is the number of individuals in OUT*i*.

Box-and-whisker plots, based on the Chao1, Shannon, and Simpson diversity indices, were constructed to visualize the α-diversity of fungi associated with apple fruit samples. Statistics were calculated using an analysis of variance (ANOVA) and the non-parametric Wilcoxon test using R software [54]. In terms of the β-diversity analyses, the data were assessed to evaluate the similarities between each condition using a correlation plot and non-metric multidimensional scaling (NMDS). The NMDS, based on the Bray–Curtis distance matrix, was applied to assess how well the derived ordination fits the given dissimilarities using Kruskal’s stress. According to Clarke [58], Kruskal’s stress values less than 0.2 represent plots with good ordination. In addition, we also used the analysis of similarity (ANOSIM) to determine the differences in fungal composition among the samples based on the presence/absence of differences according to the Jaccard similarity index [59]. The ANOSIM with 999 permutations generates an R-value ranging from 0 (completely similar) to 1 (entirely dissimilar) [60]. For these β-diversity analyses, the data were also analyzed using R software. Furthermore, the different fungal taxa were used to evaluate the different abundant taxa in each fruit part using the linear discriminant analysis (LDA) effect size (LEfSe) method with the online Galaxy workflow framework (http://huttenhower.sph.harvard.edu/lefse/ (accessed on 25 April 2020)) and an effect size threshold of 2 (logarithmic LDA score > 2) [61]. For LEfSe analysis, the alpha value for the factorial Kruskal–Wallis test was used to calculate significance among classes, and the alpha value for the pairwise Wilcoxon test between subclasses was set to 0.05.

In addition, the obtained fungal genera were used to identify the potential fungal functional groups based on the FUNGuid tool (i.e., the online guilds application tool) [62], and the relevant articles. 

## 3. Results

### 3.1. Characterization of High-Throughput Sequencing Data

**Figure 1:**
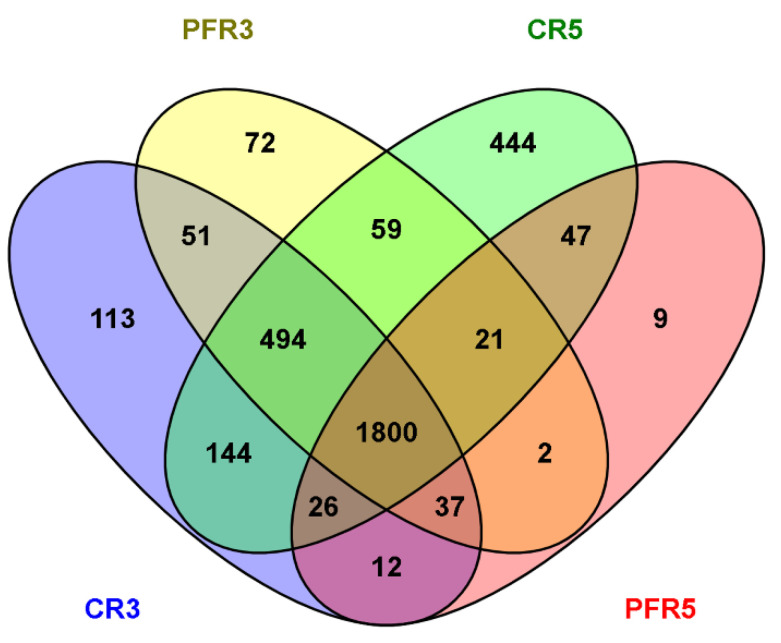
The Venn diagrams show the total number of 3331 OTUs (97% sequence identity) shared across different samples, as well as the number of OTUs present exclusively in calyx-end and peel tissues derived from bagged (i.e., CR5, PFR5) and unbagged (i.e., CR3 and PFR3) fruit.

Rare fungal OTUs, including singletons, doubletons, tripletons, and other sequences with a proportion of ≤0.001% of the total sequences (5,452,550 sequences), were filtered and removed. All obtained quality-filtered 4,967,771 sequences (91.11% of total sequences) were clustered into 3331 operational taxonomic units (OTUs), which were compared with the UNITE ITS database at ≥97% similarity. We determined the shared OTUs in the samples using a Venn diagram. All elements were shared by 1800 fungal OTUs, accounting for 54.04% of all 3331 OTUs (Figure 1). 

### 3.2. An Overall Attribution of Fungal Community Composition

A large proportion of the obtained OTUs were assigned to Ascomycota (2749 OTUs) than Basidiomycota (551 OTUs), and the rest were unidentified OTUs (31 OTUs), which accounted for 82.53%, 16.54%, and 0.93% of the total fungal OTUs, respectively. Thus, the community compositions of the fungi distributed in and on apple fruit consisted of Ascomycota, which had a relative abundance of 77.60%, followed by Basidiomycota (22.11%), and unidentified fungi (0.29%). The relative abundance of fungi distributed in these two phyla belonged to different classification levels. In general, in terms of Ascomycota, Dothideomycetes was the dominant class, accounting for 41.86% of the fungi distributed across all samples. In addition, the order Pleosporales (31.63%) and family Didymellaceae (6.20%) were the dominant groups belonging to this class. The second dominant class was Sordariomycetes, with 7.66% across all samples. The dominant class of Basidiomycota was Tremellomycetes with 15.79%, where Tremellales (15.10%) and Bulleribasidiaceae (6.47%) were the dominant order and family, respectively, belonging to this group (Figure S1).

**Figure 2:**
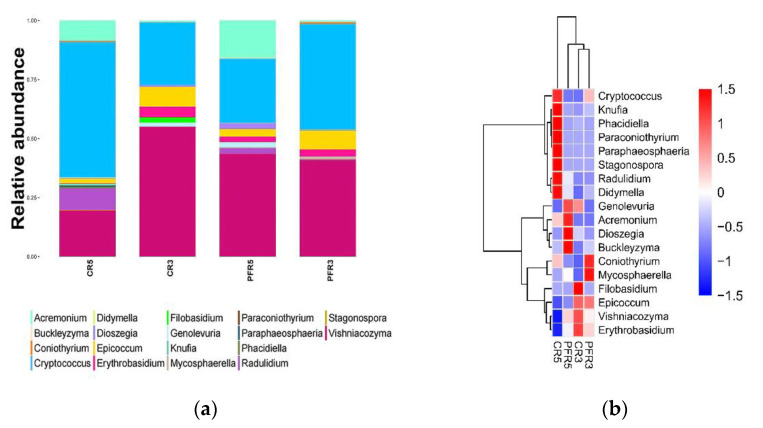
Bar charts (**a**) and heat map (**b**) showing the relative abundance of 18 fungal genera that were present across all samples. In the heat map, blue represents a fungus with relatively low abundance, and red represents a fungus with relatively high abundance. Cluster analyses of samples (Vertical) and genus classification units (horizontal) were performed according to the similarity of distributions.

In the present study, the distribution of 47 identified fungi was found across all samples of apple fruit (Table S1). As shown in Figure 2, the representative 18 fungal genera were biased in both calyx-end and peel tissues of bagged and unbagged fruit with different relative abundances. Genera *Cryptococcus*, *Knufia*, *Phacidiella*, *Paraconiothyrium*, *Paraphaeospora*, *Radulidium* and *Didymella* were mainly distributed in CR5; *Genolevuria*, *Acremonium*, *Dioszegia* and *Buckleyzyma* were mainly distributed in PFR5; *Cryptococcus*, *Coniothyrium*, *Mycosphaerella*, *Erythrobasidium* and *Epicoccum* were mainly distributed in PFR3. In CR3, the samples were mainly enriched with *Epicoccum*, *Erythrobasidium*, *Filobasidium* and *Vishniacozyma*.

### 3.3. Fungal Richness and Diversity among Apple Fruit Samples

**Figure 3:**
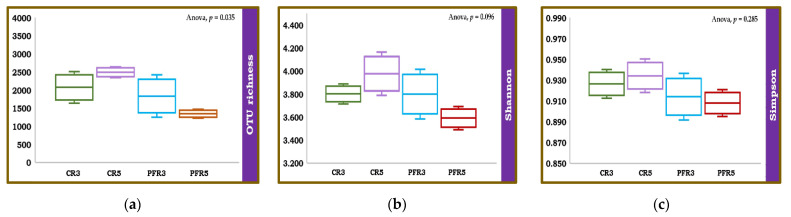
Box-and-whisker plots, based on Chao1 (**a**), Shannon (**b**), and Simpson (**c**) diversity indices showing fungal richness and diversity in the calyx-end and peel tissues derived from bagged (i.e., CR5 and PFR5) and unbagged (i.e., CR3 and PFR3) fruit. The Chao1 index is commonly used to estimate the number of OTUs or species in samples. The Shannon and Simpson diversity indices are common measures of diversity, which reflect the richness and evenness of the samples.

The number of expected reads in the single sample was approximated using rarefaction [63]. In the current study, the rarefaction curves had a fairly steep slope from the beginning to the end of the tail (Figure S2), suggesting that the sample-based rarefaction curves were not saturated; therefore, additional samples would be able to isolate more fungi. The richness and alpha diversity of fungal communities inhabiting fruit parts (i.e., calyx-end and peel) of bagged and unbagged apple fruit were estimated using the Chao1, Shannon, and Simpson indices. An analysis of variance (ANOVA) was applied to determine the means (i.e., replicates of apple fruit) of richness and alpha diversity values of fungal communities within different samples. The results showed no significant difference across samples for alpha diversity (i.e., Shannon and Simpson). In contrast, there was a significant difference in OUT richness (Chao1) of the fungal communities across apple samples (Figure 3). We then applied the student *t*-test to find the difference in fungal richness OUT among the samples. The results of the pairwise comparison showed that there was a significant difference (*p* < 0.05) between CR5 and PFR5 (Table 2).

**Figure 4:**
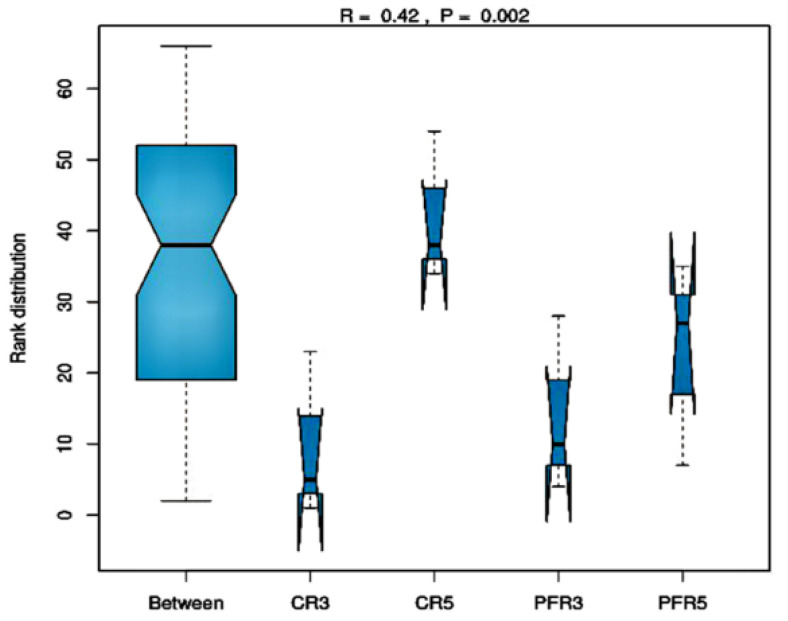
Box-and-whisker plots visualizing the results of the analysis of similarity (ANOSIM) to estimate fungal communities associated with calyx-end and peel tissues of different management practices, i.e., bagging (CR5 and PFR5) and non-bagging (CR3 and PFR3). In the plot, the *y*-axis represents the distant rank between samples, and the *x*-axis represents the results among samples.

**Figure 5:**
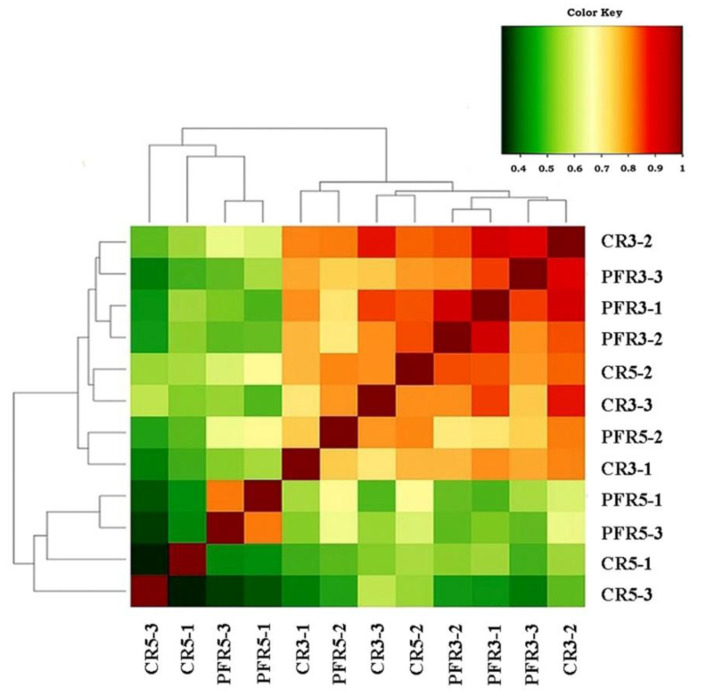
The correlation heatmap illustrates the relationship between each dataset of the calyx-end and peel tissues derived from bagged (i.e., CR5, PFR5) and unbagged (i.e., CR3 and PFR3) fruit and their replication compared to every other dataset. The red color indicates a close correlation, while the green color indicates a more distant correlation or given color key value exhibition ranging from 0 (complete distance) to 1 (complete close correlation) in the data.

**Figure 6:**
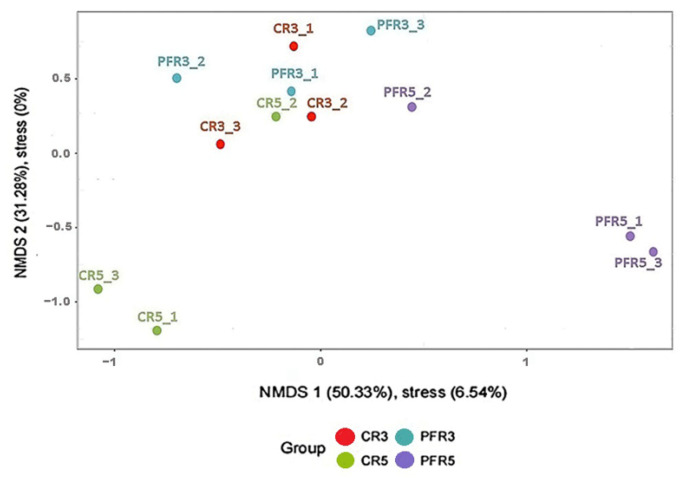
Non-metric multidimensional scale (NMDS) plots corresponding to the clustering of fungal communities in different habitats, i.e., calyx-end tissue from unbagged fruit: CR3 (red); calyx-end tissue from bagged fruit: CR5 (green); peel tissue from unbagged fruit: PFR3 (blue); peel tissue from bagged fruit: PFR5 (purple). Cluster analysis was performed using the Bray–Curtis coefficient-based community similarity measure. In the plot, the *x*-axis and *y*-axis represent the 1st and 2nd principal coordinates, respectively. The percentage of the principal coordinates represents the relative contribution of the coordinate to the sample differences. A closer distance represents higher similarity, and samples that cluster together consist of similar microbiota.

We then applied the beta diversity measure to test the differences between fungal communities from different environments. The analysis of similarity (ANOSIM) based on Jaccard’s similarity index to test whether apple fruit parts (i.e., calyx-end and peel) or management practices (i.e., bagging and non-bagging) have an impact on apple fruit inhabiting fungal communities. In terms of total detection across all samples, the ANOSIM value demonstrated differences in fungal microbiota (R = 0.420, *p* = 0.002; Figure 4). Then, analyses were separately focused. Regarding the differentiated parts of apple fruit (calyx-end versus peel), the results showed no effect of the ANOSIM value (R = 0.223, *p* = 0.068) on apple fruit parts in any of the fungal communities. At the same time, focusing on differentiated apple fruit management practices (bagging versus non-bagging) revealed a significant difference in higher ANOSIM values (R = 0.278, *p* = 0.034) of fungal communities of bagged and unbagged fruit. Consequently, it was confirmed that bagging can impact fungal communities. All samples were analyzed for these fungal similarities by generating a correlation plot and non-metric multidimensional scaling (NMDS). As shown in Figure 5, the correlation heatmap showed that most samples were red (i.e., the color key value: >0.65), indicating a close correlation, except for CR5-1, CR5-3, PFR5-1, and PFR5-3, which were more distant from most samples. With respect to CR5-1 and CR5-3, the distances were distant from one another (i.e., the color key value: <0.5), and also distant from the dataset of PFR5-1 and PFR5-3. Regarding the samples of PFR5-1 and PFR5-3, the distance was close between them (i.e., the color key value: >0.8). The NMDS was carried out to determine the similarity of fungal community compositions among different fruit parts from the different management practices. The analysis was carried out for the whole fungal community based on the Bray–Curtis coefficient index. The samples of CR5-1, CR5-3, PFR5-1, and PFR5-3 were significantly different from most of the other samples (Figure 6). Therefore, the results of these studies indicate that bagging-based management practice seem to have a profound impact on the fungal microbiota of apple fruit.

### 3.4. Analysis of Fungal Variation between Calyx-End and Peel from Bagging- and Non-Bagging-Based Practices

A metagenomic biomarker discovery approach (LEfSe) was mainly focused on the size of the effect of each differentially abundant taxon as a biomarker. The metagenomic LEfSe analyses display the cladogram and the linear discriminant analysis (LDA) coupled with effect size (LEfSe) measurements to support high-dimensional taxa comparisons. The LEfSe results were used to assess the overall fungal population in and on apple fruit. The dominant fungi in bagged fruit belonged to Ascomycota and Basidiomycota, with the same proportion of 40.74%, followed by unidentified fungi, accounting for 18.52% (Figure S3a). In contrast, the unbagged apple fruit was mainly occupied by Ascomycota, followed by Basidiomycota, accounting for 80% and 20%, respectively (Figure S3b).

**Figure 7:**
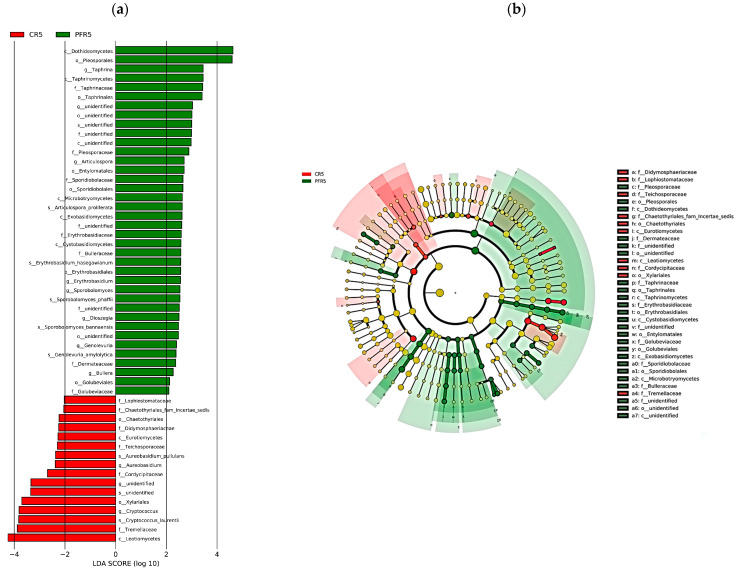
LEfSe analysis illustrating the differences in the relative abundance of taxa of calyx-end and peel tissues of bagged apple fruit. (**a**) The linear discriminant analysis (LDA) histogram exhibits biomarkers of fungal microbiota of bagged apple fruit. The histogram shows 54 taxonomic clads of fungal taxa with significant differences (cut-off score ≥ 2.0). (**b**) The cladogram with significantly discriminant taxon nodes colored. Red and green circles mean that the taxa showed differences in relative abundance, and yellow circles mean non-significant differences; branch areas are shaded according to the highest ranked group for that taxon.

Regarding bagging-based management, the LDA effect size with a cut-off score of 2.0 or greater showed that the identified abundant taxa are different on the calyx-end and peel of bagged apple fruit. There were 54 different taxa on those two apple fruit parts (Figure 7a). We observed that 38 taxa were enriched in PFR5, consisting of six classes (including unidentified class) such as Cytobasidiomycetes, Dothideomycetes, Exobasidiomycetes, Microbotryomycetes, Taphrinomycetes, and an unidentified class; eight orders (including unidentified class) such as Golubeviales, Entylomatales, Erythrobasidiales, Pleosporales, Sporidiobolales, Taphrinales, and two unidentified orders; ten families, consisting of Bulleraceae, Dermateaceae, Erythrobasidiaceae, Golubeviaceae, Pleosporaceae, Sporidiobolaceae, Taphrinaceae, and unidentified families; eight genera, consisting of *Articulospora*, *Bullera*, *Dioszegia*, *Erythrobasidium*, *Genolevuria*, *Sporobolomyces*, *Taphrina*, and an unidentified genus; six species, consisting of *Articulospora* *proliferata*, *Erythrobasidium* *hasegawianum*, *Genolevuria* *amylolytica*, *Sporobolomyces bannaensis*, *Sporobolomyces phaffii*, and unidentified species. A total of 16 taxa predominated in CR5, consisting of two classes such as Eurotiomycetes and Leotiomycete; two orders such as Chaetothyriales and Xylariales; six families such as Cordycipitaceae, Didymosphaeriaceae, Lophiostomataceae, Teichosporaceae, Tremellaceae, and Chaetothyriales fam *Incertae sedis*; three genera such as *Aureobasidium*, *Cryptococcus,* and an unidentified genus; three species such as *Aureobasidium pullulans*, *Cryptococcus laurentii*, and an unidentified species, and the cladogram exhibited the differences in the relative abundance of 34 representative taxa at five levels of the fungal community in CR5 and PFR5. Overall, the results revealed that the fungal microbiota of the bagged apple fruit consisted of various community compositions in different parts of the fruit, and there was a higher enrichment of fungal composition in peel tissues than in calyx-end tissues (Figure 7a,b).

**Figure 8:**
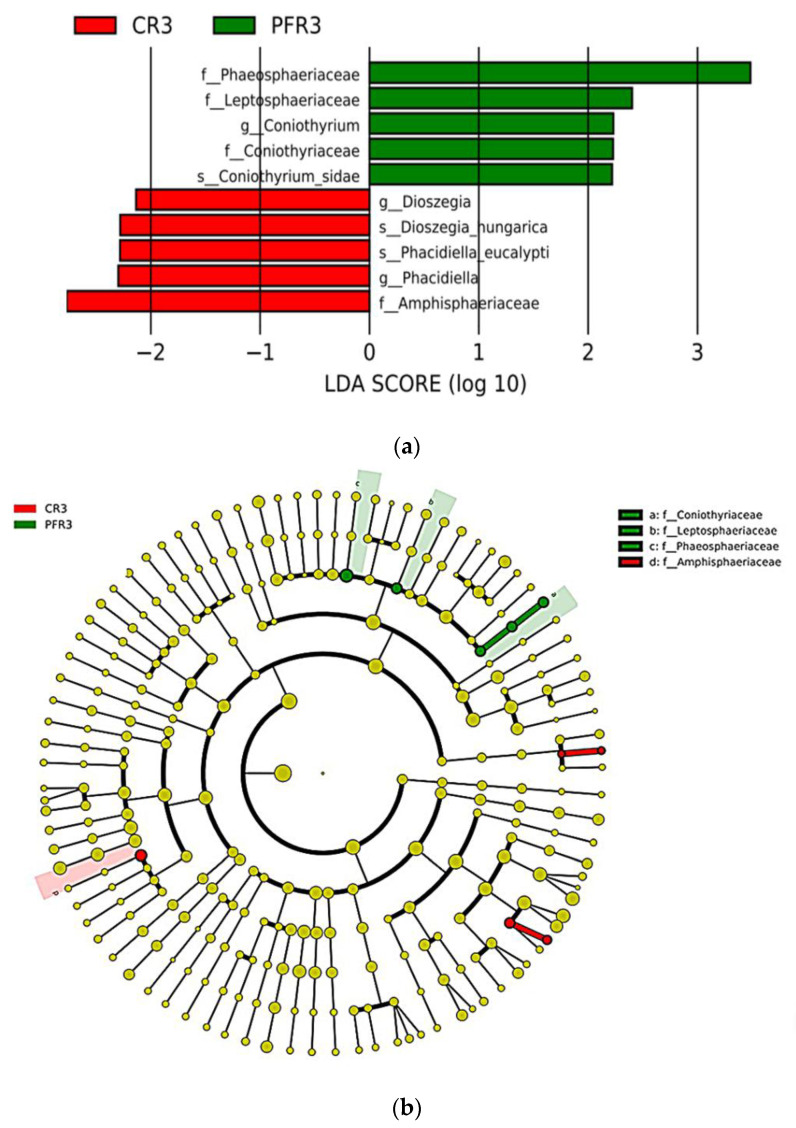
LEfSe analysis illustrating the differences in relative abundance of the taxa of calyx-end and peel tissues of unbagged apple fruit. (**a**) The linear discriminant analysis (LDA) histogram exhibits biomarkers of fungal microbiota of unbagged apple fruit. The histogram shows 10 taxonomic clades of fungal taxa with significant differences (cut-off score ≥ 2.0). (**b**) The cladogram with significantly discriminant taxon nodes colored. Red and green circles mean that taxa show differences in relative abundance, and yellow circles mean non-significant differences; branch areas are shaded according to the highest ranked group for that taxon.

Regarding non-bagging-based practice, the results showed low enrichment of fungal microbiota in the unbagged apple fruit (Figure 8a,b). There were only 10 taxa showing a significant relative abundance in the unbagged apple fruit. Phaeosphaeriaceae, Leptosphaeriaceae, Coniothyriaceae, *Coniothyrium*, and *Coniothyrium sidae* were observed in the peel, while Amphisphaeriaceae, *Dioszegia*, *Phacidiella*, *Dioszegia nungarica*, and *Phacidiella eucalypti* were observed in the calyx-end.

### 3.5. Fungal Traits of Bagged Apple Microbiome

**Figure 9:**
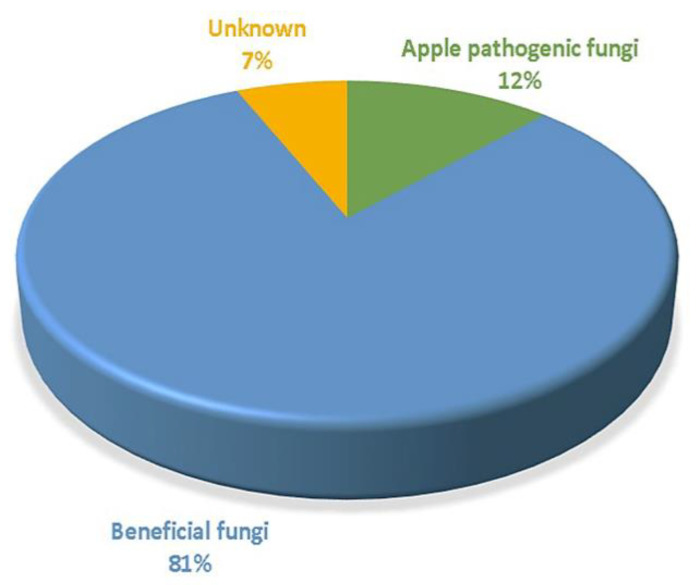
Relative abundance (%RA) at the genus level of fungal traits of apple-inhabiting fungi associated with bagged fruit. Fungal property: apple pathogenic fungi (AP), beneficial fungi (BF), unknown (U).

In the present study, we used the relative abundance of fungi at the genus level to investigate whether the fungi living in bagged apple fruit have beneficial or detrimental traits. According to fungal traits (i.e., beneficial fungi, apple pathogens, and unidentified fungi), the fungal communities from bagged fruit were mainly dominated by beneficial fungi, followed by pathogenic apple fungi and unidentified fungi, respectively (Figure 9). The potential functional properties of the fungal genera found are described in more detail in Table S2.

## 4. Discussion

A total of 3331 OTUs were assigned to taxonomic groups, with a 97% sequence similarity threshold for fungi. There was over 50% of shared OTUs, accounting for most fungal species distributed across all samples. Overall, the apple fruit-inhabiting fungal communities across all samples were mainly composed of Ascomycota followed by Basidiomycota. Ascomycota is the largest phylum of the fungal kingdom with over sixty-four thousand known fungal species [64]. Ascomycota generally has a rapid growth rate and can survive in difficult conditions, such as low nutrient levels. They can adapt to extensive substrates in challenging environments, such as water, UV, and high-temperature stresses [65,66,67]. In contrast, Basidiomycota plays a role in the degradation of plant litter with a high lignin content [68]. This likely explains why Ascomycota were the most abundant phylum in apple fruit with 77.60%, followed by Basidiomycota with 22.11%. We also found that Dothideomycetes and Sordariomycetes were significant classes belonging to Ascomycota. Several fungal pathogens causing apple diseases are in these classes [69,70,71,72,73,74,75,76]. For example, *Alternaria* [70,75] and *Epicoccum* [70] belonging to Dothideomycetes have been described as causal agents of moldy core in apple. *Acremonium*, belonging to the Sordariomycetes, is the causal agent of black and brown spot diseases [71,72]. In addition, some of the genera found in the current study (i.e., *Bullera*, *Candida,* and *Cryptococcus*) have been described as yeasts belonging to the Tremellomycetes and may play an important role as biological control agents [23,77,78,79].

Overall, the fungi were distributed in all plant parts of the apple fruit, as shown by the heatmap depicting distributions of representative 18 genera in all samples (Figure 2a,b). In general, fungi can switch their trophic mode when conditions change. Many aggressive plant pathogenic fungi can switch from biotrophic mode to saprotrophic mode living on decaying plant material to survive [36,80,81]. The most common fungal genera obtained in the current study have also been described with different functional lifestyles. For example, Buckleyzyma, Cryptococcus, Dioszegia, Filobasidium, Knufia, and Vishniacozyma are saprophytic yeasts that can live permanently on plant hosts and change their lifestyle as decomposers for survival [20,82,83,84]. In addition, *Cryptococcus*, *Filobasidium*, and *Vishniacozyma* are yeasts that have the ability to induce plant resistance [20,22,85]. Some genera such as *Acremonium* [70,72], *Coniothyrium* [70], *Didymella* [86], *Epicoccum* [70], *Paraconiothyrium* [69], and *Phacidiella* [87] have been described as apple pathogens. In addition, some genera have been frequently described as human pathogenic fungi, e.g., *Knufia* [88,89] and *Cryptococcus* [90,91]. However, some fungi commonly distributed across all samples in the current study have been rarely reported previously, e.g., *Erythrobasidium*, *Genolevuria*, and *Radulidium*. 

Regarding the composition of the fungal communities, despite several fungi being unevenly distributed in each sample, most fungi were observed in all samples. We hypothesized that the reasons for this intraspecific compositional diversity were an influence of production practices and fungal habitats. Here, we analyzed the fungal communities in the calyx-end and peel tissues in the different microenvironments of bagged and unbagged fruit. The overall diversity was high, with approximately 3000 fungal OTUs. The fungal OTU richness was higher at the calyx-end than that in the peel, both in bagged and unbagged fruit. Since the calyx-end and peel samples in the present study were composed of external and internal tissues, they would be the habitats of the internal and/or external tissue-inhabiting microorganisms [19,20]. Some microorganisms can be killed by ultraviolet (UV) irradiation [92,93]. Therefore, the calyx-end plays a crucial role by providing a source of water and nutrients for these microorganisms and protecting them from UV radiation [20]. This might be the reason why fungal OUT richness was higher in the calyx-end than that in the peel. Regarding the assessment of alpha diversity of the fungal population in apple fruit, neither the Shannon nor the Simpson diversity index showed significant results across all samples. In contrast, the Chao1 estimator showed significant differences in the bagged fruit tissue samples (i.e., CR5 and PFR5), likely indicating that the microenvironments inside the bag were responsible for the differences in fungal richness conditions. Similarly, a previous study revealed that the various climate conditions in different habitats have effects on the abundance and richness of fungi [94]. Although this current study did not show a significant difference in the alpha diversity of fungal communities within each sample, the calyx-end samples had higher diversity than the peel samples. This result is similar to that of a previous study by Abdelfattah (2020), which showed significantly higher fungal diversity in the calyx-end than in the peel tissue of apple fruit [39]. As to the sample-based rarefaction curves not being saturated, additional sampling would likely isolate more fungi, leading to highlighting the alpha diversity aspect [63,95].

In terms of beta diversity assessment of apple-inhabiting fungi, ANOSIM, NMDS and a correlation heat map demonstrated whether production practices or even parts of the apple fruit have an impact on apple-inhabiting fungal communities. The ANOSIM results showed that management practices had a significantly strong influence on the spatial distance of fungal communities, but no influence was found for fruit parts. In addition, NMDS and a correlation plot showed remarkable results: most of the fungal communities in the bagged samples were distant from the large closer communities. These results confirmed that fruit bagging has a strong influence on the community structure of fungi in apple fruit. According to a previous study, bagging can enable the fruit surface to maintain high relative humidity (RH) [72]. The microclimatic conditions inside bags change depending on the time; during the night or rain, the RH inside the bags is increased accordingly [96]. Since temperature and humidity can affect the growth of fungi [94], the conditions inside the bags might be directly responsible for differences in community composition, abundance, and richness of fungi in apples, especially when they occur in different parts of the apple fruit. Based on the present study, a distant correlation between CR5-1 and CR5-3 and a close correlation between PFR5-1 and PFR5-3 were shown, indicating that the fruit parts under bagging conditions also had effects on fungal communities.

LEfSe analysis was able to discriminate at least a single clade for statistically significant biomarkers [61]. In the current study, LEfSe was applied to identify discriminating taxa between different apple fruit parts and different management practices. LEfSe analysis showed that the number of biomarkers was higher in the bagged sample than the unbagged sample. The reasons for the different fungal communities in each niche (bagged and unbagged samples) could be due to either the influence of the pesticide or the omission of fruit bagging since we conducted a management program (Table 1) by spraying bagged and unbagged apple fruit with pesticides. Pesticides with contact and systemic properties can persist in and on apple tissues [19] and apparently suppress target plant pathogens. Although pesticides can control the pathogens and insects concerned, they also affect non-target microorganisms, which include potentially beneficial fungi [19,97,98]. In general, an ecosystem reaches stability and is healthy when it contains many species or a diverse population [23]. Our results showed an overall fungal community with low richness and low abundance in unbagged fruit. Accordingly, pesticides may directly affect the fungal population in unbagged fruit. This suggests that pesticide use poses a threat to the health of the ecosystem (phytosphere), as microorganisms are an important component of the ecosystem [23]. At the same time, the abundance and richness of fungal populations were higher in bagged fruit, so the bag can directly protect the apple fruit and the microorganisms living in fruit from pesticides. Bagging counteracted the loss of fungal microorganisms and reduced chemical residues on apple. This result is consistent with previous research findings in that fruit bagging can reduce disease and prevent pesticide residues [12,59]. Furthermore, this suggests that bagging-based management practice leads to a more diverse community.

Shen et al. [23] reported that a healthier environment could provide a more diverse fungal community, and this viewpoint is also consistent with other previous reports [19,99]. Moreover, a more diverse microbial population is mainly composed of beneficial microorganisms [19]. Similar results were achieved in the present study. The dominant composition of the fungal microbiota of bagged apple fruit was investigated by LEfSe analysis. We found a total of 54 differentially abundant taxonomic taxa. Most of the taxa present in the peel tissues of bagged apple fruit, such as the genera *Articulospora* [100], *Bullera* [78], *Dioszegia* [101], *Erythrobasidium* [102], and *Sporobolomyces* [82,102], have been reported to exhibit antifungal activity or growth-promoting activity, whereas the genus *Taphrina* has been reported to play an essential role as a pathogenic fungus, for example, *T. deformans* is the causative agent of peach leaf curl [103,104]. In addition, Dothideomycetes (particularly Pleosporaceae) were revealed as the most abundant biomarker in peel tissues. Previous studies reported that several potential apple pathogens belong to this class [70,75]. Therefore, it was very likely that various apple pathogenic fungi were enriched in the peel tissues of the bagged apple fruit. However, it has also been reported that many fungal species of this class play an important role as biological control agents against pathogenic fungi associated with various plant hosts [105,106,107,108], including apple [109,110,111,112,113]. Several taxa observed on the peel of the bagged fruit have been shown to be potential biocontrol agents (Table S2), suggesting that the peel is a favorable habitat for beneficial fungi. Therefore, peel tissue is probably the most suitable part for isolating potential biological control agents.

Regarding the calyx-end, two dominant genera, *Aureobasidium* and *Cryptococcus*, were significantly enriched in such fruit parts. The genus *Aureobasidium* has been described as a causal agent of apple diseases. *A. pullulans* is the predominant species of this genus that was significantly distributed in calyx-end in the current study. The yeast-like A. pullulans have been found on fruit surfaces and leaves of apples, which, as causal agents of russeting, caused the death of epidermal and hypodermal tissue [114]. They have been reported to invade the floral parts of apple fruit during blooming [69,115] and under humid conditions [116,117], e.g., high humidity in bags [118], which are favorable for their growth. This could be the reason that *A. pullulans* was most abundant in the calyx-end tissues of bagged fruit. In addition, *Aureobasidium* spp. (e.g., *A*. *pullulans*) have been shown to be effective biocontrol agents against various postharvest and field diseases on different plants [102,111,112,113,119]. Other dominant fungi belonging to Tremellaceae (especially the yeasts *Cryptococcus* spp.) have also been reported to exhibit a potentially antagonistic property [30,120]. *C. laurentii*, the predominant species, has been reported to be capable of enhancing plant growth as a biofertilizer for plants in nature [121]. Moreover, many fungal species in other classes (e.g., Eurotiomycetes and Leotiomycete) and families (e.g., Cordycipitaceae, Didymosphaeriaceae) have also been reported to play an important role as biological control agents [100,122,123,124]; at the same time, some of them have also been reported to show function as pathogenic fungi [125,126,127,128]. In addition, approximately 18% of the taxonomic clades of the total biomarkers were shown in the LDA histogram (Figure 7a) that were not assigned to any known taxa. One reason is that ITS regions have high inter- and intra-specific variability; the taxonomic assignments at the generally agreed threshold of 97% similarity for fungi are unable to identify some genetically related species [129]. Another reason might be that only minimal fungal species were sequenced, and their sequences were uploaded to GenBank. This suggests that these are new or unknown species in the bagged apple, and such fungal microbiota are worthy of further investigation.

Regarding the fungal microbiota in the unbagged fruit, only a small number of taxa were found in both calyx-end and peel samples. These biomarkers are potential antagonists and pathogenic fungi. The predominant biomarkers in the calyx-end were Phaeosphaeriaceae (particularly *Coniothyrium*). The genus *Coniothyrium* has been described as a causal agent of several diseases [130,131], such as moldy core of apple fruit [131]. Some *Coniothyrium* species have been described as a parasite of fungus *Sclerotinia sclerotiorum*, a widespread pathogen causing yield losses in many economic crops [132] including apple [133]. In terms of peel tissues, the genera *Diozegia* (particularly *D. nungarica*) and *Phacidiella* (particularly *P. eucalypti*) were enriched in such fruit parts and have been described as protective fungi [134] and pathogenic fungi [87], respectively. 

The plant microbiota consists of microorganisms that are potentially helpful, harmful, and neutral. In healthy plants, members of the plant-associated microbial communities coexist readily. Individual members of microbial microbiota may possess certain helpful traits [135]. Thus, many studies have reported that healthy communities are mainly occupied by beneficial microorganisms [19,23]. This is in agreement with the present LEfSe study showing that there was a high ratio of taxa with beneficial traits compared to those with pathogenic traits, especially in bagging conditions. This means that fruit bagging can greatly increase the diversity of beneficial microorganisms. On the other hand, we expected that the increase in beneficial fungi might also contribute to the reduction in diseases for bagged apples, in addition to the physical block to pathogens due to bagging. In addition, we observed potentially yield-increasing and plant-promoting fungi in bagged fruit (Table S2). This means that apples are rich reservoirs of fungal resources for developing biocontrol, yield-increasing, and plant-promoting agents.

Interestingly, the significant fungi that were enriched in the unbagged fruit were members of the Ascomycota. The Ascomycota fungi are highly adaptable. Hence, they can survive in varied environments [65,66,67]. Thus, it is not surprising to find them in an environment with chemical pollutants. The current study revealed that those fungi belonging to Ascomycota dominated over other fungi, including Basidiomycota, in unbagged fruit (Figure S3b). This study indicated that ecological equilibrium might be decreased both in apples and orchards. On the other hand, the apple microbiome of bagged fruit reached a healthy biodiverse balance due to being composed of equal OTU richness of Ascomycota and Basidiomycota (Figure S3a). The reason for the enrichment of Basidiomycota in bagged fruit was that these fungi are strict aerobes, possessing a diverse metabolic potential that drives them to survive and adapt well to the phytobiome [136]. 

Either Ascomycota or Basidiomycota was likely an important driver in the microenvironment in fruit bagging conditions. Plant hosts and several fungal strains belonging to Ascomycota and Basidiomycota shaped complex mutualistic relationships. In general, these fungal microorganisms can produce bioactive compounds that play a significant role in the physiological activities of plants, supporting mechanisms to promote plant growth and plant protection [137,138]. For example, anthocyanin is a bioactive compound in various plants [139,140,141,142,143]. The accumulation of anthocyanins in apples has also been studied with respect to the effects of bagging on the fruit quality of apples [11]. The effect of anthocyanins on fungal communities is reported in several plant fruits [144,145,146], and its effect on fungal communities in bagged apple conditions is an interesting topic for future research. This study confirmed that fruit bagging affects the richness, composition, and diversity of the fungal communities in apple fruit. However, more detailed information on the fungal community structure influenced by bagging-based practice towards microclimatic conditions and other abiotic stress factors, as well as the corresponding ecological interactions, requires further investigation.

## 5. Conclusions

The current study examined the effects of different management practices (i.e., bagging- and non-bagging-based practices) and fungal communities associated with different parts of the apple fruit (e.g., calyx-end and peel). Although the limited genetic variation within the analyzed barcode gene (ITS regions) did not allow the precise identification of several discovered taxa, the multivariate analysis provided a deeper insight into the apple-associated fungal communities. The results showed that the influence of management practices on fungal community structure was more significant than the influence of apple fruit parts. Although the fungal community was not significantly affected by their habitats, the apple fruit parts influenced the differentiation of fungal communities under bagging conditions. Fruit bagging-based management practice impacted the fungal abundance, richness, beta-diversity, and the composition of the fungal communities in and on apple fruit compared to non-bagging-based management practice. We speculate that the microclimate under bagging conditions increases the preponderance of beneficial fungi and promotes the diversity of fungal communities. Therefore, fruit bagging-based management practice not only improve the quality of apples but also maintains healthier fungal communities. Apples are rich reservoirs of fungal resources for developing biocontrol, yield-increasing, and plant-promoting agents. This study provides a foundation for understanding the effects of bagging-based management practice on associated fungal microbiota.

## Figures and Tables

**Table 1:** Management of the spray program using pesticides for pest and disease control used in the current study to evaluate the effects of bagging (R5)- and non-bagging (R3)-based management practices on the microbiota of apple fruit.

Application Date in 2019	Associated Pesticides
10 May	30% pyraclostrobin • tebuconazole + 23% lambda-cyhalothrin
25 May	30% pyraclostrobin • tebuconazole + 12% deltamethrin • thiamethoxam
20 June	30% hexaconazole + 70% propsenzine + 2.3% emamectin benzoate + 5% potassium dihydrogen phosphate
10 July	43% tebuconazole + 80% mancozeb + 23% lambda cyhalothrin + 5% potassium dihydrogen phosphate
5 August	25% propiconazole + thiophanate methyl + 12% deltamethrin • thiamethoxam + 5% potassium dihydrogen phosphate
20 August	27% thiophenone • tebuconazole + 2.3% emamectin benzoate

**Table 2:** Comparison of abundance-based estimator of OUT richness according to Chao1 index in different samples of calyx-end and peel tissues derived from bagged (i.e., CR5 and PFR5) and unbagged (i.e., CR3 and PFR3) fruit. Green boxes contain the values of mean and standard deviation determined for each apple part. White boxes contain *p*-values of the results of pairwise comparisons using the Student *t*-test.

	CR3	CR5	PFR3	PFR5
CR3	2100 ± 427	0.191	0.603	0.05
CR5	-	2510 ± 149	0.13	0.0005
PFR3	-	-	1870 ± 565	0.223
PFR5	-	-	-	1390 ± 118

## Data Availability

Not applicable.

## Supplementary Materials

The following are available online at https://www.mdpi.com/article/10.3390/jof7090764/s1, Figure S1:Bar charts showing the relative abundance of fungi distributed in class (a), order (b), and family (c) across all samples of the calyx-end and peel tissues derived from bagged (i.e., CR5 and PFR5) and unbagged (i.e., CR3 and PFR3) fruit, Figure S2: Rarefaction curves of fungi derived from different samples of apple fruit (calyx-end and peel tissues from bagged (i.e., CR5 and PFR5) and unbagged (i.e., CR3 and PFR3) fruit). The *x*-axis shows the number of valid sequences per sample, and the *y*-axis shows the observed OTUs, Figure S3: Pie charts showing the percentage of significant members (number of fungal taxa) of Ascomycota, Basidiomycota, and an unidentified phylum represented in the linear discriminant analysis (LDA) histogram of bagged (a) and unbagged (b) samples, Table S1:Analysis data of apple fruit microbiome showing forty-seven genera and forty species with their most abundant OTUs, trophic modes, growth morphology, and the percentage of relative abundances of fungal genera in the CR5, CR3, PFR5, and PFR3 samples, as well as putative genus functions detected from barcode-type next-generation sequencing, Table S2: Functions/activities of fungal genera detected in apple fruit determined by next-generation sequencing. Information is presented based on the functions of putative biological control agent (BCA) and pathogenic agent associated with apples.
