# Supplementary material for: Variations in the Community Structure of Fungal Microbiota Associated with Apple Fruit Shaped by Fruit Bagging-Based Practice
Source: J Fungi (Basel). 2021 Sep 15;7(9):764. doi: 10.3390/jof7090764 (PMC8470174; doi:10.3390/jof7090764)
Supplement: Supplementary file 1 [file jof-07-00764-s001.zip › jof-1359114-supplementary.pdf]

Variations in Community Structure of Fungal Microbiota Associated with Apple Fruit Shaped by Fruit Bagging-based practice

Punda Khwantongyim[1], Somying Wansee[1] and Xi Lu[1], Wei Zhang[1], Guangyu Sun[1,*]

[1] State Key Laboratory of Crop Stress Biology in Arid Areas, College of Plant Protection, Northwest A&F University, Yangling 712100, China

* Correspondence: sgy@nwsuaf.edu.cn

a)

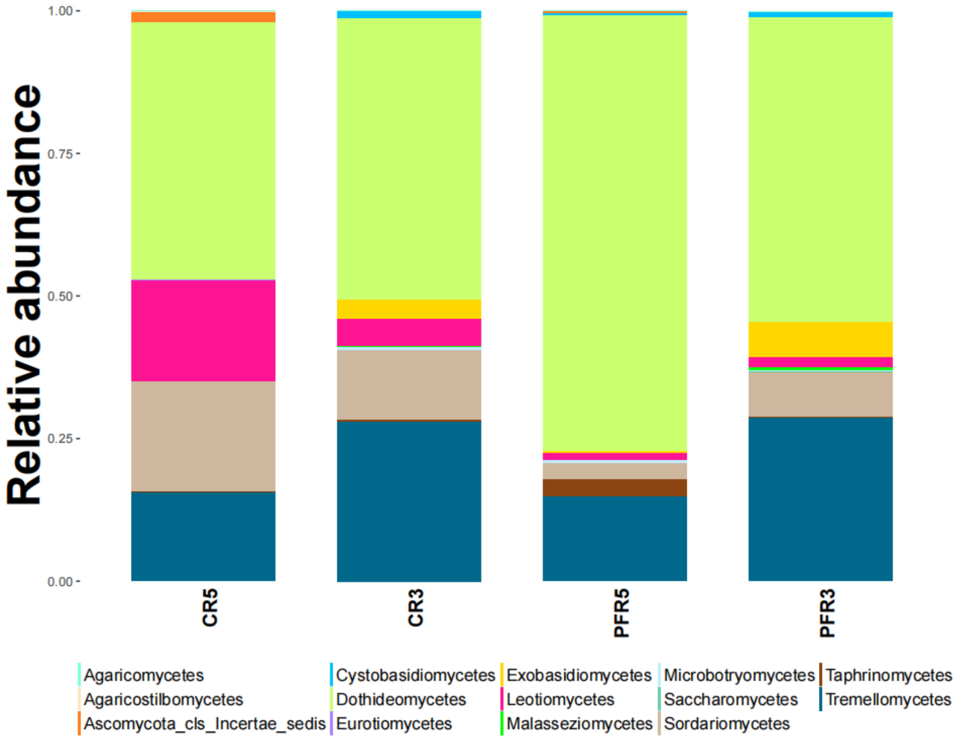
1.00
0.75
0.50
0.25
0.00
Relative abundance
CR5
CR3
PFR5
PFR3
Agaricomycetes
Agaricostilbomycetes
Ascomycota_cls_Incertae_sedis
Cystobasidiomycetes
Dothideomycetes
Eurotiomycetes
Exobasidiomycetes
Leotiomycetes
Malasseziomycetes
Microbotryomycetes
Saccharomycetes
Sordariomycetes
Taphrinomycetes
Tremellomycetes

b)

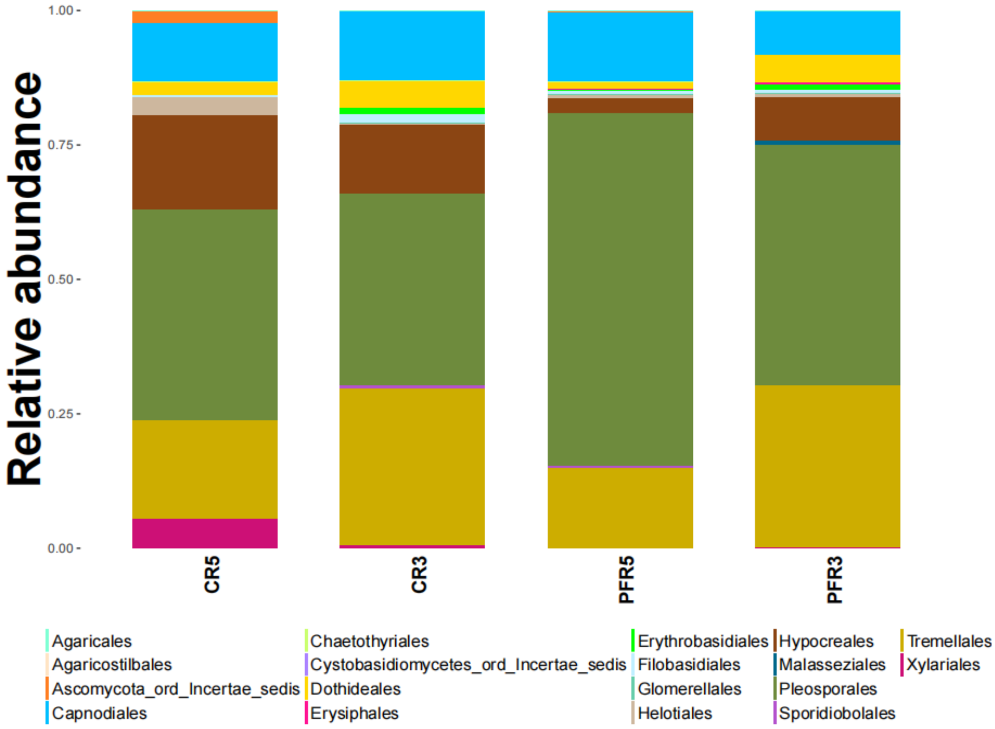
1.00
0.75
0.50
0.25
0.00
Relative abundance
CR5
CR3
PFR5
PFR3
Agaricales
Agaricostilbales
Ascomycota_ord_Incertae_sedis
Capnodiales
Chaetothyriales
Cystobasidiomycetes_ord_Incertae_sedis
Dothideales
Erysiphales
Erythrobasidiales
Filobasidiales
Glomerellales
Helotiales
Hypocreales
Malasseziales
Pleosporales
Sporidiobolales
Tremellales
Xylariales

c)

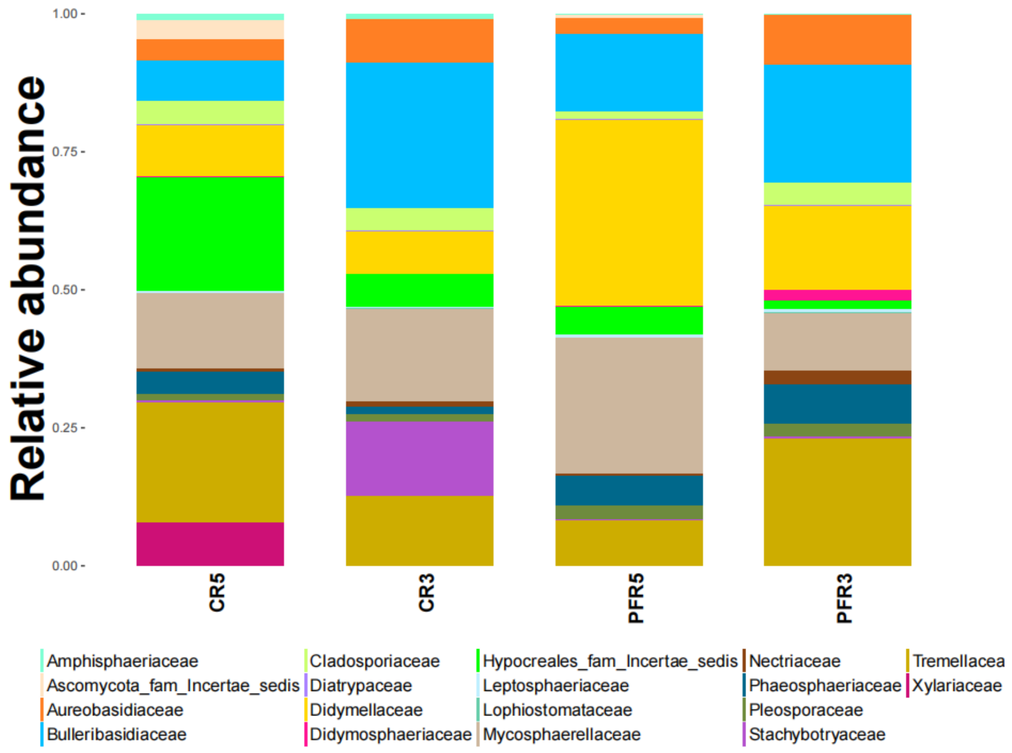

Figure S1: Bar charts showing the relative abundance of fungi distributed in class (a), order (b), and family (c) across all samples of the calyx-end and peel tissues derived from bagged (i.e., CR5, PFR5) and unbagged (i.e., CR3 and PFR3) fruit.

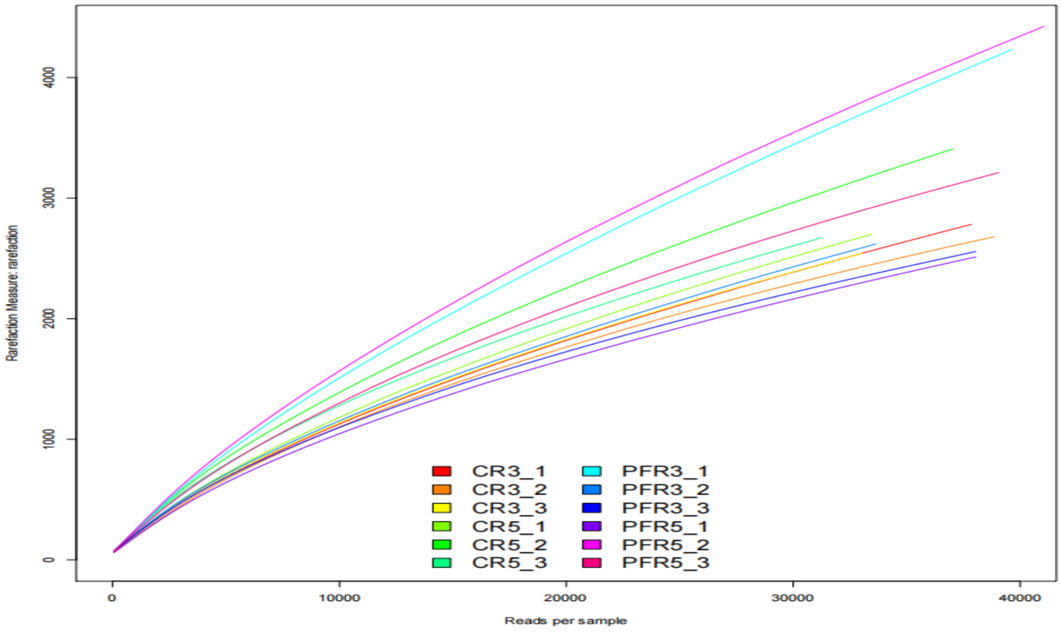

Figure S2: Rarefaction curves of fungi derived from different samples of apple fruit (calyx-end and peel tissues from bagged (i.e., CR5, PFR5) and unbagged (i.e., CR3 and PFR3) fruit). The x-axis shows the number of valid sequences per sample, and the y-axis shows the observed OTUs.

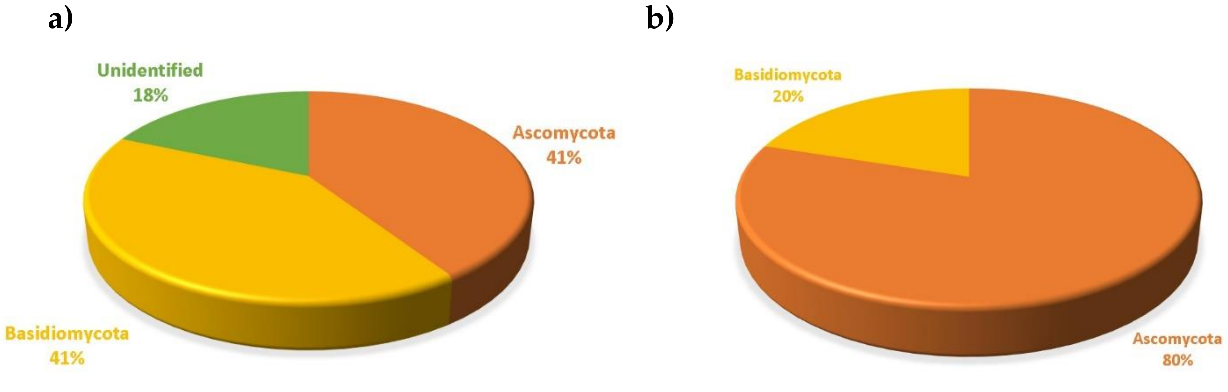

Figure S3: Pie charts showing the percentage of significant members (number of fungal taxa) of Ascomycota, Basidiomycota, and an unidentified phylum represented in the linear discriminant analysis (LDA) histogram of bagged (a) and unbagged (b) samples.

Table S1: Analysis data of apple fruit microbiome showing forty-seven genera and forty species with their most abundant OTUs, trophic modes, growth morphology, and the percentage of relative abundances of fungal genera in the CR5, CR3, PFR5, and PFR3 samples, as well as putative genus functions detected from barcode-type next-generation sequencing.

Genera/ most abundant OTUs	Identified species/ most abundant OTUs	Trophic modes	Growth morphology	Relative abundance (%)				Putative genus functions		References
				CR5	CR3	PFR5	PFR3	Biocontrol agent (BCA)	Pathogen	
Acremonium OTU_15289 (0.6639)	*Acremonium alternatum* OTU_91310 (0.0079), *Acremonium fusidioides* OTU_1203 (0.0031), *Acremonium hyalinulum* OTU_113180 (0.0033), *Acremonium rutilum* OTU_69035 (0.0021)	E, P, S	Filamentous fungi	1.1430	0.1256	1.8852	0.1125	√	√	[1–6]
Alternaria OTU_3861 (0.001578)		E, P, S	Filamentous fungi	0.0005	0.0003	0.0019	0.0019	√	√	[6–11]
Articulospora OTU_57849 (0.0558)	*Articulospora proliferata* OTU_57849 (0.0558)	E, S	Filamentous fungi	0.0005	0.0062	0.1785	0.0379	√		[12]
Aureobasidium OTU_45119 (0.0012)	*Aureobasidium pullulans* OTU_45119 (0.0012)	E, P, S	Yeast-like fungi	0.0012	0.0050	0.0000	0.0019	√	√	[13–15]
Biappendiculispora OTU_8962 (0.0066)	*Biappendiculispora japonica* OTU_8962 (0.0066)	S	Filamentous fungi	0.0000	0.0028	0.0000	0.0237			
Blumeria OTU_36017 (0.0057)	*Blumeria graminis* OTU_36017 (0.0057)	P	Filamentous fungi	0.0003	0.0001	0.0024	0.0199		√	[16,17]
Buckleyzyma OTU_4088 (0.0056)	*Buckleyzyma aurantiaca*	S	Yeast	0.0073	0.0053	0.0212	0.0106	√		[6]

	OTU_4088 (0.0056)									
Bullera<br>OTU_17277 (0.0455)		E, P, S	Yeast	0.0068	0.0180	0.1031	0.0648	√		[18,19]
Candida<br>OTU_10908 (0.0073)	*Candida hyderabadensis*<br>OTU_10908 (0.0073)	E, P, S	Yeast	0.0005	0.0087	0.0078	0.0122	√		[6,15,20]
Cercospora<br>OTU_24918 (0.0053)		E, P, S	Filamentous fungi	0.0029	0.0002	0.0163	0.0019		√	[21,22]
Coniothyrium<br>OTU_14646 (0.0410)	*Coniothyrium sidae*<br>OTU_14646 (0.0410)	E, P, S	Filamentous fungi	0.0553	0.0149	0.0175	0.1140	√	√	[6,23–26]
Cryptococcus<br>OTU_17375 (4.9685)	*Cryptococcus laurentii*<br>OTU_17375 (4.9685),<br>*Cryptococcus statzelliae*<br>OTU_20854 (0.0010),<br>*Cryptococcus uniguttulatus*<br>OTU_12263 (0.0064)	E, P, S	Yeast	7.6804	5.0146	3.2410	7.8437	√		[6,14,27]
Cystobasidium<br>OTU_10867 (0.0034)		E, P	Yeast	0.0023	0.0003	0.0019	0.0091	√		[28]
Didymella<br>OTU_18485 (0.0098)		E, P, S	Filamentous fungi	0.0432	0.0025	0.0129	0.0132	√	√	[6,29–33]
Dinemasporium<br>OTU_7667 (0.0341)		E, P, S	Null	0.0004	0.0000	0.0000	0.1417			
Dioszegia<br>OTU_4020 (0.0855)	*Dioszegia changbaiensis*<br>OTU_3401 (0.0039),<br>*Dioszegia hungarica*<br>OTU_22919 (0.0121)	E, P, S	Yeast	0.0352	0.1204	0.3091	0.0470	√		[34]
Epicoccum<br>OTU_14212 (0.8266)	*Epicoccum nigrum*<br>OTU_14212 (0.8266)	E, P, S	Filamentous fungi	0.2622	1.6004	0.3745	1.4080	√	√	[6,26,35]
Erythrobasidium<br>OTU_8827 (0.0361)	*Erythrobasidium hasegawianum*<br>OTU_8827 (0.0361)	S	Yeast	0.0246	0.8484	0.2807	0.5126	√		[15]

Filobasidium OTU_103350 (0.1041)	*Filobasidium floriforme* OTU_103350 (0.1041), *Filobasidium wieringae* OTU_8749 (0.0018)	E, S	Yeast	0.0072	0.4244	0.0028	0.0124	√		[27]
Fonsecazyma OTU_31843 (0.0009)	*Fonsecazyma mujuensis* OTU_31843 (0.0009)	U	Yeast	0.0036	0.0000	0.0000	0.0000			
Fusarium OTU_19844 (0.0051)		E, P, S	Filamentous fungus	0.0064	0.0194	0.0037	0.0089	√	√	[6,26,36]
Genolevuria OTU_8344 (0.1461)	*Genolevuria amylolytica* OTU_8344 (0.1461), *Genolevuria armeniaca* OTU_5316 (0.0009)	U	Yeast	0.0218	0.3115	0.2406	0.0359			
Knufia OTU_55479 (0.0077)	*Knufia aspidiotus* OTU_55479 (0.0077)	E, P	Filamentous fungi	0.0446	0.0002	0.0017	0.0067	√	√	[28,37]
Lachnum OTU_129208 (0.0048)	*Lachnum fuscescens* OTU_129208 (0.0048)	E, S	Filamentous fungi	0.0005	0.0001	0.0000	0.0184	√		[38]
Latorua OTU_81530 (0.0064)	*Latorua caligans* OTU_81530 (0.0064)	S	Yeast	0.0000	0.0011	0.0000	0.0244			
Lecanicillium OTU_94854 (0.0050)	*Lecanicillium primulinum* OTU_94854 (0.0050)	E, P	Filamentous fungi	0.0001	0.0000	0.0000	0.0201	√		[6,39]
Monocillium OTU_134549 (0.0058)	*Monocillium indicum* OTU_134549 (0.0058)	E, P, S	Null	0.0000	0.0000	0.0000	0.0023	√		[40]
Murilentithecium OTU_88928 (0.0139)	*Murilentithecium clematidis* OTU_88928 (0.0139)	S	Null	0.0003	0.0005	0.0000	0.0588			

Mycosphaerella OTU_26248 (0.0599)	*Mycosphaerella ellipsoidea* OTU_26248 (0.0599)	E, P, S	Filamentous fungi	0.0241	0.0055	0.0475	0.1656		√	[41–43]
Occultifur OTU_24507 (0.0016)		P, S	Yeast	0.0000	0.0063	0.0000	0.0001			
Paraconiothyrium OTU_62952 (0.0025)		E, P, S	Filamentous fungi	0.0098	0.0000	0.0000	0.0000	√	√	[44–47]
Paraphaeosphaeria OTU_94126 (0.0192)	*Paraphaeosphaeria verruculosa* OTU_94126 (0.0192)	E, P, S	Null	0.0768	0.0000	0.0000	0.0000	√		[48]
Phacidiella OTU_90178 (0.0135)	*Phacidiella eucalypti* OTU_90178 (0.0135)	P, S	Null	0.0699	0.0046	0.0000	0.0003		√	[49]
Rachicladosporium OTU_91428 (0.0015)	*Rachicladosporium inconspicuum* OTU_91428 (0.0015)	E, P, S	Filamentous fungi	0.0057	0.0001	0.0000	0.0000		√	[50]
Radulidium OTU_5956 (0.3486)	*Radulidium subulatum* OTU_5956 (0.3486)	E, S	Null	1.2310	0.0052	0.2925	0.0379			
Rhodosporidiobolus OTU_24032 (0.0167)		P, S	Filamentous fungi	0.0000	0.0630	0.0038	0.0001			
Rhodotorula OOTU_125338 (0.0050)		E, P, S	Yeast	0.0005	0.0161	0.0000	0.0034	√	√	[13,51]
Sarocladium OTU_17877 (0.0207)	*Sarocladium bactrocephalum* OTU_17877 (0.0207)	E, P, S	Filamentous fungi	0.0007	0.0111	0.0000	0.0994	√	√	[5,6,52]
Setomelanomma OTU_83222 (0.0106)		P, S	Filamentous fungi	0.0000	0.0423	0.0000	0.0000		√	[53]
Spegazzinia OTU_68436 (0.0005)		E, S	Null	0.0020	0.0000	0.0000	0.0000			
Sporobolomyces OTU_11547 (0.0408)	*Sporobolomyces bannaensis* OTU_7177 (0.0030), *Sporobolomyces phaffii* OTU_11547 (0.0408),	E, P, S	yeast	0.0024	0.0401	0.1856	0.0118	√		[15,27]

	Sporobolomyces symmetricus OTU_7042 (0.0032)									
Stagonospora OTU_93157 (0.0034)		E, P, S	Filamentous fungi	0.0137	0.0000	0.0000	0.0000		√	[54]
Stagonosporopsis OTU_32562 (0.0069)		E, P, S	Null	0.0031	0.0131	0.0000	0.0115		√	[54]
Strobilurus OTU_122869 (0.0011)		S	Filamentous fungi	0.0043	0.0002	0.0000	0.0000			
Taphrina OTU_125697 (0.0311)		E, P	Filamentous fungi	0.0017	0.2385	2.3320	0.0147	√	√	[15,55]
*Trichometasphaeri*a OTU_109962 (0.0013)		S	Null	0.0049	0.0002	0.0000	0.0000		√	[56]
Vishniacozyma OTU_30022 (3.1662)	*Vishniacozyma carnescens* OTU_29813 (1.1911), *Vishniacozyma victoriae* OTU_30022 (3.1662)	E, P, S	Yeast	2.6284	10.3875	5.1955	7.1629	√		[57]

Note: life mode —P (Pathogen), E (Endophyte), S (saprotroph), and U (Unknown)

Table S2: Functions/activities of fungal genera detected in apple fruit determined by next-generation sequencing. Information is presented based on the functions of putative biological control agent (BCA) and pathogenic agent associated with apples.

Genus	Functions/Activities		References
	**Categories of function**	**Description**	
Acremonium	Putative BCA	• *Acremonium strictum* has been proposed to serve as biocontrol agents against *Venturia inaequalis,* the causal agent of apple scab.<br>• *Acremonium alternatum* can inhibit powdery mildew of cucumber caused by *Sphaerotheca fuliginea.*<br>• *Acremonium* sp. exhibit a potential biological control of brown rot of *Nectarine Janisiewicz*.	[1–3]
	Apple pathogen	• *Acremonium* spp. causes brown and black spot disease in apple fruit.	[3,5,52]
Alternaria	Putative BCA	• *Alternaria alternata* inhibits sporulation of *Plasmopara viticola,* the causal agent of grapevine downy mildew disease.	[9]
	Apple pathogen	• *Alternari* spp. causal agent of moldy core of apple.<br>• *Alternaria alternata* f. sp. *mali* the causal agent Alternaria leaf blotch and fruit spot of apple.	[7,26,58]
Articulospora	Putative BCA	• *Articulospora* can produce antibiotic compound which can inhibit bacterial growth	[12]
	Apple pathogen	• Neutral	
Aureobasidium	Putative BCA	• *Aureobasidium pullulans* can be used as potential biocontrol agents in controlling the post-harvest decay of apples caused by *Botrytis cinerea*.<br>• *Aureobasidium pullulans* can control brown rot of nectarine fruit caused by *Monilinia fructicola.*	[3,12]
	Apple pathogen	• *Aureobasidium pullulan* is the causal agent of russet of apple fruit.	[13]
Biappendiculispora	Putative BCA	• Neutral	-
	Apple pathogen	• Neutral	
Blumeria	Putative BCA	• Neutral	-
	Apple pathogen	• Neutral	-
Buckleyzyma	Putative BCA	• *Buckleyzyma aurantiaca* impeded pathogen growth and reproduction.	[59]
	Apple pathogen	• Neutral	
Bullera	Putative BCA	• *Bullera elba* can secrete a killer toxin that inhibits the growth of some pathogenic yeasts and is most active against *Cryptococcus neoformans*.	[18]
	Apple pathogen	• Neutral	
Candida	Putative BCA	• *Candida* is found to have potential for biocontrol of brown rot of nectarines<br>• *Candida* is applied as a biocontrol of gray mold on grapes caused by *Botrytis cinerea*<br>• *Candida sake* against *Penicillium expansum* is significantly enhanced L-Serine and L-Aspartic on apples.	[15,20,60]

	Apple pathogen	• Neutral	
Cercospora	Putative BCA	• Neutral	
	Apple pathogen	• *Cercospora*, the causal agent of leaf spot disease on apple.	[61]
Coniothyrium	Putative BCA	• *Coniothyrium minitans* can control *Sclerotinia sclerotiorum*, the causal agent of the disease of lettuce and other plants.	[23,62,63]
	Apple pathogen	• *Coniothyrium* spp. the causal agent of moldy core and core rot of apple.	[64]
Cryptococcus	Putative BCA	• *Cryptococcus flavescens* can be used as potential biocontrol agents in controlling the post-harvest decay of apples caused by *Botrytis cinerea*.<br>• *Cryptococcus humicola* can control gray mold caused by *Botrytis cinerea* in apple.	[27,65,66]
	Apple pathogen	• Neutral	
Cystobasidium	Putative BCA	• *Cystobasidium* spp. increases the resistance of *Ulmus minor* to Dutch elm disease by increasing the concentration of flavonoids and phenolic compounds in xylem tissue.	[28]
	Apple pathogen	• Neutral	
Didymella	Putative BCA	• *Didymella pomorum* (*Phoma pomorum*) have been reported as biological control of damping-off disease of *Trifolium alexandrinum* L. caused by *Pythium*.	[33]
	Apple pathogen	• *Didymella pomorum*, the canker and wood rot pathogen of young apple tree.	[67]
Denemasporium	Putative BCA	• Neutral	
	Apple pathogen	• Neutral	
Dioszegia	Putative BCA	• *Dioszegia* plays an important role as biocontrol agent by supporting plant growth.	[34]
	Apple pathogen	• Neutral	
Epicoccum	Putative BCA	• *Epicoccum nigrum*, biological control agent of peach twig blight caused by *Monilinia laxa*.<br>• *Epicoccum layuense*, a potential biological control agent of esca-associated fungi in grapevine.	[68,69]
	Apple pathogen	• *Epicoccum* spp. the causal agent of moldy and core rot of apple.	[26,64]
Erythrobasidium	Putative BCA	• *Erythrobasidium* exhibits the potential as biological control agent of brown rot of nectarine.	[15]
	Apple pathogen	• Neutral	
Filobasidium	Putative BCA	• *Filobasidium* spp. have been reported their function as biological control of *Botrytis cinerea* in apple.	[27]
	Apple pathogen	• Neutral	
Fonsecazyma	Putative BCA	• Neutral	
	Apple pathogen	• Neutral	
Fusarium	Putative BCA	• *Fusarium equiseti* enhances plant growth and reduces severity of anthracnose and damping-off diseases in cucumber (*Cucumis sativus*).	[36]

	Apple pathogen	• *Fusarium* spp. have been reported as the causal agent of apple core rot.	[26]
Genolevularia	Putative BCA	• Neutral	
	Apple pathogen	• Neutral	
Knufia	Putative BCA	• *Knufia* spp. enhance *Ulmus minor* resistance to Dutch elm disease by increasing concentration of flavonoids and phenolic compounds in xylem tissues.	[28]
	Apple pathogen	• Neutral	
Lachnum	Putative BCA	• *Lachnum* isolates have the potential to enhance the growth of selected blueberry.	[38]
	Apple pathogen	• Neutral	
Latorua	Putative BCA	• Neutral	
	Apple pathogen	• Neutral	
Lecanicillium	Putative BCA	• There has been reported potential of *Lecanicillium* spp. in controlling of insects, nematodes and plant diseases.	[39]
	Apple pathogen	• Neutral	
Monocillium	Putative BCA	• *Monocillium gamsii* and *M. bulbillosum* are parasites of nematode eggs. This genus has been reported as candidates for nematode biocontrol.	[70]
	Apple pathogen	• Neutral	
Murilentithecium	Putative BCA	• Neutral	
	Apple pathogen	• Neutral	
Mycosphaerella	Putative BCA	• Neutral	
	Apple pathogen	• Neutral	
Occultifur	Putative BCA	• Neutral	
	Apple pathogen	• Neutral	
Paraconiothyrium	Putative BCA	• There have been reported ability of *Paraconiothyrium* as the antagonistic fungi against *Sclerotinia sclerotiorum* associated with *Brachiaria grasses*.	[47]
	Apple pathogen	• *Paraconiothyrium* is the causal agent of moldy core and core rot of apple.	[44]
Paraphaeosphaeria	Putative BCA	• *Paraphaeosphaeria minitans* (*Coniothyrium minitans*) control *Verticillium dahliae* the causal agent of Verticillium wilt of olive.	[48]
	Apple pathogen	• Neutral	
Phacidiella	Putative BCA	• Neutral	
	Apple pathogen	• *Phacidiella discolor* was reported as pathogenic strain associated with a dieback and canker disease of apple trees in England.	[49]
Rachicladosporium	Putative BCA	• Neutral	
Radulidium	Apple pathogen	• Neutral	
Rhodosporidiobolus	Putative BCA	• Neutral	
	Apple pathogen	• Neutral	

Rhodotorula	Putative BCA	• Rhodotorula shows its putative mechanisms of antagonism against *Botrytis cinerea*, the causative agent of apple disease.<br>• *Rhodotorula glutinis* can control postharvest pathogens on apple.<br>• *Rhodotorula* can control brown rot of nectarine fruit caused by *Monilinia fructicola*.<br>• *Rhodotorula rubra* presented antimicrobial activity and increase the growth stimulating property on the cotton seed, and protect the plant from diseases.	[15,27,71,72]
	Apple pathogen	• *Rhodotorula glutinis* the causal agent of russet on apple	[13]
Sarocladium	Putative BCA	• *Sarocladium implicatum* potential biocontrol agent of Ascochyta blight of chickpea disease caused by *Ascochyta rabiei*.	[73]
	Apple pathogen	• *Sarocladium* causes brown spot associated with bagged apple.	[5]
Setomelanomma	Putative BCA	• Neutral	
	Apple pathogen	• Neutral	
Spegazzinia	Putative BCA	• Neutral	
	Apple pathogen	• Neutral	
Sporobolomyces	Putative BCA	• *Sporobolomyces* has ability of biological control agent against brown rot of nectarine.	[15]
	Apple pathogen	• Neutral	
Stagonospora	Putative BCA	• Neutral	
	Apple pathogen	• Neutral	
Stagonosporopsis	Putative BCA	• Neutral	
	Apple pathogen	• Neutral	
Strobilurus	Putative BCA	• Neutral	
	Apple pathogen	• Neutral	
Taphrina	Putative BCA	• *Taphrina* can control brown rot of nectarine fruit caused by *Monilinia fructicola*.	[15]
	Apple pathogen	• Neutral	
Trichometasphaeria	Putative BCA	• Neutral	
	Apple pathogen	• Neutral	
Vishniacozyma	Putative BCA	• *Vishniacozyma victoriae* shows biocontrol activity against *Penicillium expansum* and *Botrytis cinerea*, the causal agents of postharvest diseases such as blue and grey mold on pear fruit.	[74]
	Apple pathogen	• Neutral	

References

1. Malathrakis, N.E. The fungus Acremonium alternatum Lin: Fr., a hyperparasite of the cucurbits powdery mildew pathogen *Sphaerotheca fuliginea*. *Zeitschrift fuer Pflanzenkrankheiten und Pflanzenschutz* **1985**, *92*, 509–515.

2. Gessler, C.; Reidy, B.; Lotscher, T.; Schloffer, K. *Acremonium strictum*: A potential antagonist of *Venturia inaequalis*. *IOBC WPRS Bullet* **2000**, *23*, 183–186.

3. Guo, Y.Z.; Sun, G.Y.; Gao, B.W.; Li, C.Y.; Zhang, P.L.; Lei, X.L. Studies on the identification of pathogen and the biological characteristics of black-dot disease of bagged apple. *Acta Agric. Boreali-occidentalis Sinia* **2005**, *14*, 18-21 (in Chinese).

4. Jayawardena, R.S.; Zhang, W.; Liu, M.; Maharachchikumbura, S.S.N.; Zhou, Y.; Huang, J.B.; Nilthong, S.; Wang, Z.Y.; Li, X.H.; Yan, J.Y.; et al. Identification and characterization of *Pestalotiopsis*-like fungi related to grapevine diseases in China. *Fungal Biol.* **2015**, *119*, 348–361, doi:10.1016/j.funbio.2014.11.001.

5. Hou, Y.M.; Zhang, X.; Zhang, N.N.; Naklumpa, W.; Zhao, W.Y.; Liang, X.F.; Zhang, R.; Sun, G.Y.; Gleason, M.L. Genera *Acremonium* and *Sarocladium* cause brown spot on bagged apple fruit in China. *Plant Dis.* **2019**, *103*, 1889–1901, doi:10.1094/PDIS-10-18-1794-RE.

6. Thambugala, K.M. Daranagama, D.A.; Phillips, A.J.L.; Kannangara, S.D. Promputtha, I. Fungi in biocontrol: An overview of fungal antagonists applied against fungal plant pathogens. *Front. Cell. Infect. Microbiol* **2020**, *10*, 604923, doi:10.3389/fcimb.2020.604923.

7. Ellis, M.A.; Barrat, J.G. Colonization of Delicious apple fruits by *Alternaria* spp. and effect of fungicide sprays on moldy-core. *Plant Dis.* **1983**, *67*, 50–152.

8. Thomma, B.P.H.J. *Alternaria* spp.: From general saprophyte to specific parasite. *Mol. Plant Pathol.* **2003**, *4*, 225–236, doi:10.1046/j.1364-3703.2003.00173.x.

9. Musetti, R.; Vecchione, A.; Stringher, L.; Borselli, S.; Zulini, L.; Marzani, C.; D'Ambrosio, M.; Di Toppi, L.S.; Pertot, I. Inhibition of sporulation and ultrastructural alterations of grapevine downy mildew by the endophytic fungus *Alternaria alternata*. *Phytopathology* **2006**, *96*, 689–698, doi:10.1094/PHYTO-96-0689.

10. Gao, Y.H.; Sun, W.; Su, Y.Y.; Cai, L. Three new species of *Phomopsis* in Gutianshan nature reserve in China. *Mycol. Prog.* **2014**, *13*, 111–121, doi:10.1007/s11557-013-0898-2.

11. Elfar, K.; Zoffoli, J.P.; Latorre, B.A. Identification and characterization of *Alternaria* species associated with moldy core of apple in Chile. *Plant Dis.* **2018**, *102*, 2158–2169, doi:10.1094/pdis-02-18-0282-re.

12. Sugahara, H.; Kondo, T.; Okada, M.; Ikeda, Y.; Kaida, K.; Fudou, R.; Mizuno, T.; Sakagami, Y. *Articulospora* sp. produces Art1, an inhibitor of bacterial histidine kinase. *Biosci. Biotechnol. Biochem.* **2008**, *72*, 2521–2525, doi:10.1271/bbb.80021.

13. Matteson Heidenreich, M.C.; Corral-Garcia, M.R.; Momol, E.A.; Burr, T.J. Russet of apple fruit caused by A*ureobasidium pullulans* and *Rhodotorula glutinis*. *Plant Dis.* **1997**, *81*, 337–342, doi:10.1094/pdis.1997.81.4.337.

14. Gildemacher, P.; Heijne, B.; Silvestri, M.; Houbraken, J.; Hoekstra, E.; Theelen, B.; Boekhout, T. Interactions between yeasts, fungicides and apple fruit russeting. *FEMS Yeast Res.* **2006**, *6*, 1149–1156, doi:10.1111/j.1567-1364.2006.00109.x.

15. Janisiewicz, W.J.; Kurtzman, C.P.; Buyer, J.S. Yeasts associated with nectarines and their potential for biological control of brown rot. *Yeast* **2010**, *27*, 389–398, doi:10.1002/yea.1763.

16. Costamilan, L.M. Variability of the wheat powdery mildew pathogen *Blumeria graminis* f. sp. *tritici* in the 2003 crop season. *Fitopatol. Bras.* **2005**, *30*, 420–422, doi:10.1590/S0100-41582005000400015.

17. Cowger, C.; Mehra, L.; Arellano, C.; Meyers, E.; Murphy, J.P. Virulence differences in *Blumeria graminis* f. sp. *tritici* from the central and eastern United States. *Phytopathology* **2018**, *108*, 402–411, doi:10.1094/PHYTO-06-17-0211-R.

18. Golubev, W.; Ikeda, R.; Shinoda, T.; Nakase, T. Antifungal activity of *Bullera alba* (Hanna) derx. *Mycoscience* **1997**, *38*, 25–29, doi:10.1007/BF02464965.

19. Guo, J.; Bowatte, S.; Hou, F. Diversity of endophytic bacteria and fungi in seeds of *Elymus nutans* growing in

four locations of Qinghai Tibet Plateau, China. *Plant and SoilSoil* **2020**, *459*, 49–63, doi:10.1007/s11104-020-04608-y.

20. Kasfi, K.; Taheri, P.; Jafarpour, B.; Tarighi, S. Identification of epiphytic yeasts and bacteria with potential for biocontrol of grey mold disease on table grapes caused by *Botrytis cinerea*. *Spanish J. Agric. Res.* **2018**, *16*, e1002, doi:10.5424/sjar/2018161-11378.

21. Skaracis, G.N.; Pavli, O.I.; Biancardi, E. Cercospora leaf spot disease of sugar beet. *Sugar Tech* **2010**, *12*, 220–228, doi:10.1007/s12355-010-0055-z.

22. Pataky, N.R. *Fungal leaf spot diseases of shade and ornamental trees in the midwest*; University of Illinois, Urbana-Champaign, 1998; Vol. RPD No 648;

23. Whipps, J.M.; Gerlagh, M. Biology of *Coniothyrium minitans* and its potential for use in disease biocontrol. *Mycol. Res.* **1992**, *96*, 897–907, doi:10.1016/s0953-7562(09)80588-1.

24. Gao, J.; Ma, L.; Li, X.; Han, J. Studies on antagonistic action of *Coniothyrium minitans* against *Sclerotinia sclerotiorum*. *Plant Prot.* **2006**, *32*, 66–70.

25. Whipps, L.M.; Sreenivasaprasad, S.; Muthumeenakshi, S.; Rogers, C.W.; Challen, M.P. Use of *Coniothyrium minitans* as a biocontrol agent and some molecular aspects of sclerotial mycoparasitism. *Eur. J. Plant Pathol.* **2008**, *121*, 323–330.

26. Gao, L.L.; Zhang, Q.; Sun, X.Y.; Jiang, L.; Zhang, R.; Sun, G.Y.; Zha, Y.L.; Biggs, A.R. Etiology of moldy core, core browning, and core rot of Fuji apple in China. *Plant Dis.* **2013**, *97*, 510–516, doi:10.1094/PDIS-01-12-0024-RE.

27. Filonow, A.B.; Vishniac, H.S.; Anderson, J.A.; Janisiewicz, W.J. Biological control of *Botrytis cinerea* in apple by yeasts from various habitats and their putative mechanisms of antagonism. *Biol. Control* **1996**, *7*, 212–220, doi:10.1006/bcon.1996.0086.

28. Martínez-Arias, C.; Sobrino-Plata, J.; Ormeño-Moncalvillo, S.; Gil, L.; Rodríguez-Calcerrada, J.; Martín, J.A. Endophyte inoculation enhances *Ulmus minor* resistance to Dutch elm disease. *Fungal Ecol.* **2021**, *50*, doi:10.1016/j.funeco.2020.101024.

29. Morgan-Jones, G.; Burch, K.B. Studies in the genus Phoma. XI. Concerning *Phoma lycopersici*, the anamorph of *Didymella lycopersici*, causal organism of stem canker and fruit rot of tomato. *Mycotaxon* **1988**, *32*, 133–142.

30. Besri, M. Control of canker caused by *Didymella lycopersici* in tomato by solar heating (solarization) of stakes. *Phytopathol. Zeitschrift* **1983**, *108*, 333–340.

31. Channon, A.G. Control of tomato stem rot (*Didymella lycopersici* Kleb.) with benomyl. *Hortic. Res.* **1972**, *12*, 89–96.

32. El-Mahjoub, M. Foot rot of *Solanum sodomaeum* caused by *Didymella lycopersici*. *Plant Dis. Report.* *63*, 431.

33. Maghazy, S.M.N.; H.M.A., A.; Haridy, M.S.; Moustafa, S.M.N. Biological control of damping-off disease of *Trifolium alexandrinum* L.caused by *Pythium spinosum* Sawada var. *spinosum* using some soil fungi. *Arch. Phytopathol. Plant Prot.* **2008**, *41*, 431–450, doi:10.1080/03235400600813680.

34. Araujo, R.; Dunlap, C.; Franco, C.M.M. Analogous wheat root rhizosphere microbial successions in field and greenhouse trials in the presence of biocontrol agents *Paenibacillus peoriae* SP9 and *Streptomyces fulvissimus* FU14. *Mol. Plant Pathol.* **2020**, *21*, 622–635, doi:10.1111/mpp.12918.

35. Wittig, H.P.P.; Johnson, K.B.; Pscheidt, J.W. Effect of epiphytic fungi on brown rot blossom blight and latent infections in sweet cherry. *Plant Dis.* **1997**, *81*, 383–387, doi:10.1094/PDIS.1997.81.4.383.

36. Saldajeno, M.G.B.; Hyakumachi, M. The plant growth-promoting fungus *Fusarium equiseti* and the arbuscular mycorrhizal fungus *Glomus mosseae* stimulate plant growth and reduce severity of anthracnose and damping-off diseases in cucumber (*Cucumis sativus*) seedlings. *Ann. Appl. Biol.* **2011**, *159*, 28–40, doi:10.1111/j.1744-7348.2011.00471.x.

37. Hutchison, L.J.; Untereiner, W.A.; Hiratsuka, Y. *Knufia cryptophialidica* gen. et sp. nov., a dematiaceous hyphomycete isolated from Black Galls of Trembling Aspen (*Populus tremuloides*). *Mycologia* **1995**, *87*, 902, doi:10.2307/3760868.

38. Bizabani, C.; Dames, J. Effects of inoculating *Lachnum* and *Cadophora* isolates on the growth of *Vaccinium corymbosum*. *Microbiol. Res.* **2015**, *181*, 68–74, doi:10.1016/j.micres.2015.08.005.

39. Goettel, M.S.; Koike, M.; Kim, J.J.; Aiuchi, D.; Shinya, R.; Brodeur, J. Potential of *Lecanicillium* spp. for management of insects, nematodes and plant diseases. *J. Invertebr. Pathol.* **2008**, *98*, 256–261, doi:10.1016/j.jip.2008.01.009.

40. Ashrafi, S.; Stadler, M.; Dababat, A.A.; Richert-Pöggeler, K.R.; Finckh, M.R.; Maier, W. *Monocillium gamsii* sp. nov. and *Monocillium bulbillosum*: Two nematode-associated fungi parasitising the eggs of Heterodera filipjevi. *MycoKeys* **2017**, *27*, 21–38, doi:10.3897/mycokeys.27.21254.

41. Dabek, A.J.; Waller, J.M. Black leaf streak and viral leaf streak: new banana diseases in East Africa. *Trop. Pest Manag.* **1990**, *36*, 157–158.

42. Carlier, J.; Zapater, M.F.; Lapeyre, F.; Jones, D.R.; Mourichon, X. Septoria leaf spot of banana: a newly discovered disease caused by *Mycosphaerella eumusae* (anamorph *Septoria eumusae*). *Phytopathology* **2000**, *90*, 884–890.

43. Arzanlou, M.; Abeln, E.C.A.; Kema, G.H.J.; Waalwijk, C.; Carrier, J.; De Vries, I.; Guzman, M.; Crous, P.W. Molecular diagnostics for the Sigatoka disease complex of banana. *Phytopathology* **2007**, *97*, 1112–1118, doi:10.1094/PHYTO-97-9-1112.

44. Michailides, T.J.; Morgan, D.P.; Mitcham, E.; Crisosto, C. Occurrence of mouldy core and core rot of Fuji apple in California. *KAC Plant Prot. Q.* **1994**, *3*, 4–6.

45. Verkley, G.J.M.; Da Silva, M.; Wicklow, D.T.; Crous, P.W. *Paraconiothyrium*, a new genus to accommodate the mycoparasite *Coniothyrium minitans*, anamorphs of *Paraphaeosphaeria*, and four new species. *Stud. Mycol.* **2004**, *50*, 323–335.

46. Damm, U.; Verkley, G.J.M.; Crous, P.W.; Fourie, P.H.; Haegi, A.; Riccioni, L. Novel *Paraconiothyrium* species on stone fruit trees and other woody hosts. *Persoonia* **2008**, *20*, 9–17, doi:10.3767/003158508X286842.

47. Gama, D.D.S.; Santos, Í.A.F.M.; De Abreu, L.M.; De Medeiros, F.H.V.; Duarte, W.F.; Cardoso, P.G. Endophytic fungi from *Brachiaria grasses* in Brazil and preliminary screening of *Sclerotinia sclerotiorum* antagonists. *Sci. Agric.* **2020**, *77*, e20180210, doi:10.1590/1678-992x-2018-0210.

48. Varo, A.; Raya-Ortega, M.C.; Trapero, A. Selection and evaluation of microorganisms for biocontrol of *Verticillium dahlia* in olive. *J. Appl. Microbiol* **2016**, *121*, 767–777, doi:10.1111/jam.13199.

49. Brooks, F.T. On the occurrence of *Phacidiella discolor* (Mout. & Sacc.) Potebnia in England. *Trans. Br. Mycol. Soc.* **1928**, *13*, 75–81, doi:10.1016/s0007-1536(28)80005-2.

50. Matteson Cheewangkoon, R.; Groenewald, J.Z.; Summerell, B.A.; Hyde, K.D.; To-anun, C.; Crous, P.W. Myrtaceae, a cache of fungal biodiversity. *Persoonia Mol. Phylogeny Evol. Fungi* **2009**, *23*, 55–85, doi:10.3767/003158509X474752.

51. Raspor, P.; Miklič-Milek, D.; Avbelj, M.; Čadež, N. Biocontrol of grey mould disease on grape caused by *Botrytis cinerea* with autochthonous wine yeasts. *Food Technol. Biotechnol.* **2010**, *48*, 336–343.

52. Wang, C.C.; Jin, J.; Li, B.H.; Wang, C.X.; Dong, X.L.; Zhang, Z.F. Pathogens isolated from different symptoms of black spot disease and infection conditions. *Acta Agric. Boreali Sin.* **2014**, *29*, 136–144.

53. Rossman, A.Y.; Farr, D.F.; Castlebury, L.A.; Shoemaker, R.; Mengistu, A. *Setomelanomma holmii* (pleosporales, phaeosphaeriaceae) on living spruce twigs in Europe and North America. *Can. J. Bot.* **2002**, *80*, 1209–1215.

54. Garibaldi, A.; Bertetti, D.; Ortu, G.; Gullino, M.L. A Leaf Spot caused by *Stagonosporopsis trachelii* on *Campanula medium* in Italy. *J. Plant Pathol.* **2015**, *97*, 546.

55. Cissé, O.H.; Almeida, J.M.G.C.F.; Fonseca, Á.; Kumar, A.A.; Salojärvi, J.; Overmyer, K.; Hauser, P.M.; Pagni, M. Genome sequencing of the plant pathogen *Taphrina deformans*, the causal agent of peach leaf curl. *MBio* **2013**, *4*, 1–8, doi:10.1128/mBio.00055-13.

56. Mitiku, M.; Eshte, Y.; Shiferaw, W. Evaluation of maize variety for northern leaf blight (*Trichometasphaeria turcica)* in South Omo zone. *World J. Agric. Res.* **2014**, *2*, 237–239, doi:10.12691/wjar-2-5-6.

57. Gramisci, B.R.; Lutz, M.C.; Lopes, C.A.; Sangorrín, M.P. Enhancing the efficacy of yeast biocontrol agents

against postharvest pathogens through nutrient profiling and the use of other additives. *Biol. Control* **2018**, *121*, 151–158, doi:10.1016/j.biocontrol.2018.03.001.

58. Gur, L.; Reuveni, M.; Cohen, Y. Occurrence and etiology of Alternaria leaf blotch and fruit spot of apple caused by *Alternaria alternata* f. sp. *mali* on cv. Pink lady in Israel. *Eur. J. Plant Pathol.* **2017**, *147*, 695–708, doi:10.1007/s10658-016-1037-0.

59. Charlie, P.; Tania, F.; Agnès, C.; Julie, F. d'Arcier; Emilie, C.; Massot, M.; Julien, C.; Stéphane, R.; David A., B.; Jessica, V.; et al. Microbial association networks give relevant insights into plant pathobiomes. *bioRxiv* **2019**, doi:10.1101/2020.02.21.958033.

60. Nunes, C.; Usall, J.; Teixidó, N.; Miró, M.; Viñas, I. Nutritional enhancement of biocontrol activity of *Candida sake* (CPA-1) against *Penicillium expansum* on apples and pears. *Eur. J. Plant Pathol.* **2001**, *107*, 543–551, doi:10.1023/A:1011288710875.

61. Thapa, R.; Zhang, K.; Snavely, N.; Belongie, S.; Khan, A. The plant pathology challenge 2020 data set to classify foliar disease of apples. *Appl. Plant Sci.* **2020**, *8*, e11390, doi:10.1002/aps3.11390.

62. Jones, E.E.; Whipps, J.M. Effect of inoculum rates and sources of *Coniothyrium minitans* oncontrol of *Sclerotinia sclerotiorum* disease in glasshouse lettuce. *Eur. J. Plant Pathol.* **2002**, *108*, 527–538, doi:10.1023/A:1019940820230.

63. De Vrije, T.; Antoine, N.; Buitelaar, R.M.; Bruckner, S.; Dissevelt, M.; Durand, A.; Gerlagh, M.; Jones, E.E.; Lüth, P.; Oostra, J.; et al. The fungal biocontrol agent *Coniothyrium minitans*: production by solid-state fermentation, application and marketing. *Appl. Microbiol. Biotechnol.* **2001**, *56*, 58–68, doi:10.1007/s002530100678.

64. Grove, G.G.; Eastwell, K.C.; Jones, A.L.; Sutton, T.B. Diseases of apple. In *Apples: botany, production and uses*; Ferree, D.C., Warrington, I.J., Eds.; CAB International: Wallingford, USA, 2003; pp. 459–488.

65. Kheireddine, A.; Essghaier, B.; Hedi, A.; Dhieb, C.; Sadfi-Zouaoui, N. New epiphytic yeasts able to reduce grey mold disease on apples. *Plant Prot. Sci.* **2018**, *54*, 248–257.

66. Gezahgne, A.; Cortinas, M.N.; Wingfield, M.J.; Roux, J. Characterisation of the Coniothyrium stem canker pathogen on *Eucalyptus camaldulensis* in Ethiopia. *Australas. Plant Pathol.* **2005**, *34*, 85–90, doi:10.1071/AP04079.

67. Havenga, M.; Gatsi, G.M.; Halleen, F.; Spies, C.F.J.; van der Merwe, R.; Mostert, L. Canker and wood rot pathogens present in young apple trees and propagation material in the Western Cape of South Africa. *Plant Dis.* **2019**, *103*, 3129–3141, doi:10.1094/PDIS-04-19-0867-RE.

68. Madrigal, C.; Pascual, S.; Melgarejo, P. Biological control of peach twig blight (*Monilinia laxa*) with *Epicoccum nigrum*. *Plant Pathol.* **1994**, *43*, 554–561, doi:10.1111/j.1365-3059.1994.tb01590.x.

69. Del Frari, G.; Cabral, A.; Nascimento, T.; Ferreira, R.B.; Oliveira, H. *Epicoccum layuense* a potential biological control agent of esca-associated fungi in grapevine. *PLoS One* **2019**, *14*, 1–22, doi:10.1371/journal.pone.0213273.

70. Ashrafi, S.; Stadler, M.; Dababat, A.A.; Richert-Pöggeler, K.R.; Finckh, M.R.; Maier, W. *Monocillium gamsii* sp. nov. and *Monocillium bulbillosum*: Two nematode-associated fungi parasitising the eggs of *Heterodera filipjevi*. *MycoKeys* **2017**, *27*, 21–38, doi:10.3897/mycokeys.27.21254.

71. Zhang, H.; Wang, L.; Ma, L.; Dong, Y.; Jiang, S.; Xu, B.; Zheng, X. Biocontrol of major postharvest pathogens on apple using *Rhodotorula glutinis* and its effects on postharvest quality parameters. *Biol. Control* **2009**, *48*, 79–83, doi:10.1016/j.biocontrol.2008.09.004.

72. Akhtyamova, N.; Sattarova, R.K. Endophytic yeast *Rhodotorula rubra* strain TG-1: Antagonistic and plant protection activities. *Biochem. Physiol. Open Access* **2013**, *2*, 1–5, doi:10.4172/2168-9652.1000104.

73. Rajakumar, E.; Aggarwal, R.; Singh, B. Fungal antagonists for the biological control of Ascochyta blight of chickpea. *Acta Phytopathol. Entomol. Hungarica* **2005**, *40*, 35–42, doi:10.1556/APhyt.40.2005.1-2.5.

74. Gramisci, B.R.; Lutz, M.C.; Lopes, C.A.; Sangorrín, M.P. Enhancing the efficacy of yeast biocontrol agents against postharvest pathogens through nutrient profiling and the use of other additives. *Biol. Control* **2018**, *121*, 151–158, doi:10.1016/j.biocontrol.2018.03.001.
